# Mechanisms of In Vitro Cytotoxicity of Honeybee Venom Components and Melittin-Functionalized Fe_3_O_4_ Nanoparticles on HaCaT Keratinocytes and A375 Melanoma Cells

**DOI:** 10.3390/ma19153260

**Published:** 2026-08-01

**Authors:** Alex Câmpian, Olga Sorițău, Mara Muntean, Cristian Iacoviță, Oana Baldasici, Otilia Andercou, Adrian Florea

**Affiliations:** 1 Department of Cell and Molecular Biology, Faculty of Medicine, “Iuliu Hațieganu” University of Medicine and Pharmacy, 6 Louis Pasteur St., 400349 Cluj-Napoca, Romania; alexcampian@yahoo.com (A.C.); muntean.mara@elearn.umfcluj.ro (M.M.); aflorea@umfcluj.ro (A.F.); 2 Radiobiology and Tumor Biology Laboratory, The Oncology Institute “Prof. Dr. Ion Chiricuță”, 34–36 Republicii St., 400015 Cluj-Napoca, Romania; olgasoritau@yahoo.com; 3 Department of Pharmaceutical Physics-Biophysics, Faculty of Pharmacy, “Iuliu Hațieganu” University of Medicine and Pharmacy, 6 Louis Pasteur St., 400349 Cluj-Napoca, Romania; cristian.iacovita@umfcluj.ro; 4 Department of Genetics, Genomics and Experimental Pathology, The Oncology Institute “Prof. Dr. Ion Chiricuță”, 34–36 Republicii St., 400015 Cluj-Napoca, Romania; oana_baldasici@yahoo.ro

**Keywords:** bee venom, melittin-functionalized magnetic nanoparticles, A375 cells, HaCaT cells, magnetic hyperthermia, apoptosis, antitumor activity

## Abstract

Melittin (Mel), the principal cytolytic peptide in honeybee venom (BV), has anticancer activity but limited selectivity. This study examined whether adsorption of Mel to Fe_3_O_4_ magnetic nanoparticles (MNPs and MNPs-Mel, respectively), alone or combined with magnetic hyperthermia (MH), modifies cytotoxicity and cell-death phenotype in A375 melanoma cells relative to HaCaT keratinocytes. BV, free Mel, and phospholipase A_2_ (PLA) were first screened by Alamar Blue assay to select dose-matched conditions; MNPs-Mel (50 or 100 µg/mL MNPs, equivalent to approximately 3 or 6 µg/mL Mel) was then compared with dose-matched free Mel and unfunctionalized MNPs, and the MNP-containing groups were additionally evaluated with MH. Outcomes included metabolic activity, Annexin V/propidium iodide flow cytometry, transmission electron microscopy (TEM), and clonogenic potential. At the lower dose, MNPs-Mel produced a larger Annexin-positive fraction in A375 than in HaCaT cells (28.30% vs. 11.80%) and a lower viable-cell fraction (71.64% vs. 87.55%); relative to dose-matched free Mel, the A375 response shifted toward early apoptosis. After MNPs-Mel plus MH, too few A375 cells remained for reliable cytometric acquisition, whereas HaCaT populations remained quantifiable and showed 25.56–29.45% apoptosis, predominantly late apoptosis. Free PLA produced smaller changes in metabolic activity than BV or Mel at composition-matched concentrations. TEM supported nanoparticle internalization and treatment-associated mitochondrial and lysosomal alterations. These findings suggest that MNP association changes the cellular presentation of low-dose Mel, while MH increases overall treatment intensity but may narrow the separation between malignant and nonmalignant cells.

## 1. Introduction

Cutaneous melanoma is a malignant neoplasm arising from melanocytes and is one of the most aggressive forms of skin cancer because of its capacity for invasion and metastatic dissemination. According to GLOBOCAN 2022, approximately 331,700 new melanoma cases and 58,700 associated deaths occurred worldwide, while the estimated five-year prevalence exceeded 1.26 million cases [1]. The incidence varies considerably among geographical regions and is highest in fair-skinned populations, particularly in Australia and New Zealand, Europe, and North America. Ultraviolet radiation is the principal modifiable risk factor. If incidence and mortality rates remain unchanged, the global burden has been projected to reach approximately 510,000 new cases and 96,000 deaths annually by 2040 [2]. The management of cutaneous melanoma depends primarily on disease stage, tumor thickness, ulceration, lymph-node involvement, and molecular characteristics. Wide local excision represents the principal treatment for localized melanoma, with sentinel lymph-node biopsy used for staging in selected patients. Immune-checkpoint inhibitors targeting PD-1 and CTLA-4 have substantially improved outcomes in high-risk and advanced melanoma, while combined BRAF and MEK inhibition represents an important option for tumors harboring a BRAF V600 mutation. Surgery, radiotherapy, and other local treatments may also be used in selected locoregional or metastatic cases [3]. Nevertheless, primary or acquired treatment resistance, recurrence, immune-related toxicity, and the absence of durable responses in some patients continue to support the investigation of new therapeutic strategies.

A first step in testing potential candidates as antitumor molecules is experimentation in vitro on simple models. For melanoma studies, a well-characterized and widely used cell line is A375, derived from a human primary malignant melanoma. These cells have an epithelial morphology and are hypotriploids, with 62 chromosomes. In vivo, they can form rapidly growing tumors, with an amelanotic melanoma appearance, especially in the case of immunosuppression [4,5].

On the other hand, HaCaT cells can be used as control and reference cells in comparing the cell-dependent effects of the newly tested active agents. They are spontaneously immortalized, aneuploid human keratinocytes derived from adult skin. Although these cells possess unlimited proliferative capacity, they remain non-tumorigenic and retain several features of epidermal differentiation, making them a reproducible nonmalignant keratinocyte model for studying skin biology and treatment-associated toxicity [6]. However, HaCaT cells have undergone spontaneous transformation and contain ultraviolet-associated TP53 mutations; consequently, they should not be considered fully equivalent to primary human keratinocytes or normal skin tissue [7].

Natural products are an important source of biologically active molecules and have contributed substantially to the development of antimicrobial, anti-inflammatory, analgesic, and anticancer agents. Among animal-derived products, bee venom (BV) has attracted considerable scientific interest because it contains a complex mixture of compounds with diverse biological activities. Bee venom composition and its biological activity may vary according to bee species and subspecies, geographical origin, season, and venom-collection method [8,9,10]. The dry weight of honeybee (particularly of *Apis mellifera*) venom consists predominantly of peptides and proteins. Melittin (Mel) is its most abundant component and generally represents 50–55% of the dry venom, whereas phospholipase A_2_ (PLA) is the principal enzymatic molecule and commonly accounts for approximately 10–12%. Other constituents include proteins (hyaluronidase, acid phosphatase, α-glucosidase, and dipeptidyl peptidase IV), peptides (apamin, mast-cell-degranulating peptide, adolapin, secapin, tertiapin, and procamine peptides), amino acids, histamine, dopamine, and noradrenaline, along with sugars, phospholipids, and volatile substances [8,9,10,11]. Consequently, the complex effects of whole BV reflect the combined, and sometimes synergistic, actions of several components, especially Mel and PLA [12,13].

Melittin is a linear, cationic peptide composed of 26 amino acids, and it has a molecular weight of approximately 2.85 kDa. It possesses a well-characterized membrane-disruptive action that accounts for much of its general cytotoxic activity (as discussed below). At sufficiently high concentrations, extensive bilayer destabilization produces rapid ion leakage, osmotic imbalance, swelling, loss of membrane integrity, and cellular lysis. Direct microscopic examination of gastric and colorectal cancer cells has demonstrated that Mel can cause rapid membrane blebbing, swelling, surface disruption, and concentration-dependent cell death within a few hours [14]. These findings are important when interpreting cell-death outcomes because high Mel concentrations may induce predominantly lytic or necrotic injury rather than a slowly developing classical apoptotic process, as reviewed in [15]. At lower or intermediate concentrations, however, membrane perturbation by Mel may initiate intracellular stress responses before irreversible lysis occurs. Reported effects include calcium influx, inner mitochondrial membrane depolarization, generation of reactive oxygen species, cytochrome-c release, alteration of the BAX/BCL-2 balance, caspase activation, and cleavage of apoptosis-associated substrates. Depending on the cancer model, Mel has also been associated with inhibition of phosphoinositide 3-kinase/protein kinase B/mammalian target of rapamycin signaling, mitogen-activated protein kinase pathways, nuclear factor kappa B activation, Janus kinase/signal transducer and activator of transcription signaling, and pathways involved in migration and invasion [16,17,18,19].

Experimental evidence supports the antitumoral activity of Mel in several cancer models [20,21]. In melanoma cells, BV and Mel reduced proliferation and migration and altered PI3K/AKT/mTOR and MAPK signaling [17]. Melittin obtained from *A. florea* venom decreased A375 melanoma-cell viability and promoted Annexin V-positive populations together with changes in apoptosis-associated gene expression [18]. Dose- and time-dependent apoptosis has also been observed in human leukemia cells [19]. In aggressive breast cancer models, BV and Mel induced rapid cell death and interfered with growth-factor receptor signaling, particularly in HER2-enriched and triple-negative cells [22]. Additional studies have reported induction of death-receptor and mitochondrial apoptotic pathways in ovarian cancer cells and inhibition of Rac1-associated migration in hepatocellular carcinoma [23,24]. It was recently demonstrated by Câmpian et al. that Mel reduced metabolic viability and ATP levels and altered LDH and caspase-9 responses in Caco-2 colorectal adenocarcinoma cells [25]. These results demonstrate that Mel can affect both the plasma membrane and intracellular signaling. Nevertheless, the relative contribution of these mechanisms depends on dose and exposure time. Low exposure may permit the development of regulated cell-death pathways, whereas higher concentrations may produce membrane damage so rapidly that apoptosis-associated events are obscured by necrosis, secondary necrosis, detachment, or cellular fragmentation [15].

Despite its antitumoral potential, free Mel has major therapeutic limitations. Due to its relatively generalized affinity for cell membranes, its membrane activity is not intrinsically restricted to malignant cells, and exposure of normal tissues can result in hemolysis, nonspecific cytotoxicity, inflammation, and vascular damage. Free Mel may also undergo proteolytic degradation and rapid interaction with serum proteins and cell membranes, limiting its stability and effective distribution. On the other hand, when administered in low doses to avoid the undesired side effects on healthy cells, the efficacy of the antitumor treatments with Mel is reduced. These disadvantages have encouraged the development of liposomal, polymeric, lipid-based, protein-based, and inorganic delivery systems designed to reduce premature membrane contact, improve peptide stability, and favor accumulation or controlled release at the target site [16,26,27,28].

One strategy for improving the delivery of membrane-active compounds—Mel in our particular case—involves magnetic nanoparticles (MNPs). Iron oxide MNPs, particularly magnetite with the chemical formula Fe_3_O_4_, are among the most extensively investigated inorganic materials in biomedical nanotechnology. Fe_3_O_4_ MNPs have been studied for magnetic resonance imaging, magnetic separation, cell tracking, biosensing, magnetically guided drug delivery, and magnetic hyperthermia. The possibility of combining diagnostic and therapeutic functions within the same platform has led to their description as theranostic materials [29,30]. Their biodegradation can release iron that enters physiological iron-processing pathways, but excessive intracellular accumulation may disturb iron homeostasis, promote reactive oxygen species formation, and contribute to oxidative injury. Toxicity is therefore dependent on dose, coating, aggregation state, cellular localization, exposure duration, and the capacity of the cell to process the released iron [30,31]. An interesting function of Fe_3_O_4_ MNPs speculated in our study is their role as carriers for biologically active peptides. Surface conjugation of MNPs with Mel (MNPs-Mel) offers the possibility of reducing the freely diffusible peptide fraction, modifying cellular uptake, and concentrating the membrane-active compound in association with the nanoparticle. This is why it is recommended to use lower but still effective doses on the targeted cells, without too many side effects. Nevertheless, conjugation does not automatically guarantee tumor selectivity or safety. A formulation must therefore be compared with equivalent concentrations of free Mel, unconjugated MNPs, and untreated controls [15,16,25]. Another advantage of Fe_3_O_4_ MNPs is represented by their possible delivery by magnetic guidance to the site of the tumor. Finally, the MNPs have the capacity to generate heat when exposed to an alternating magnetic field (AMF)—the basis of magnetic hyperthermia (MH). We have previously reported the ability of Mel-functionalized Fe_3_O_4_ MNPs to reduce metabolic viability and ATP levels, and to increase LDH and caspase-9 responses, while their combination with MH produced a stronger overall antitumor effect [25,32].

In the present study, we aimed to evaluate the antitumor effects of low doses of whole BV, free Mel and PLA, as well as their dose-dependent effects. To further elucidate the intracellular molecular mechanisms responsible for the anticancer activity of Mel, MNPs-Mel were also tested at different doses, with or without MH contribution. These experimental settings could help us distinguish the contributions of peptide-mediated membrane damage, phospholipid hydrolysis, nanoparticle-associated effects, and heat-induced cytotoxicity. Comparative analysis in malignant (A375) and nonmalignant (HaCaT) cellular models was particularly important for determining whether increased antitumoral activity is accompanied by an acceptable degree of selectivity. Because BV, Mel, PLA, and MNPs-Mel and MH can each produce different cell-death phenotypes depending on treatment intensity and exposure time, our results were based on measurements of metabolic viability, absolute cell recovery, DNA integrity, apoptosis, necrosis, cellular ultrastructure, and proliferation disposition.

## 2. Materials and Methods

### 2.1. Honeybee Venom, Melittin, and Phospholipase A_2_

The crystallized BV was collected by A.F. from *A. mellifera* bees, as previously described [33]. Lyophilized Mel purified from BV (product number: M2272, molecular weight: 2846.46 Da, and purity: 96%), and lyophilized PLA purified from BV (product number: P9279, molecular weight: 14,500 Da, and purity: 1956.57 units/mg protein) were obtained from Sigma-Aldrich (St. Louis, MO, USA). BV was stored at 4 °C, and 24 h before the experimental treatment, it was dissolved in bidistilled water at a concentration of 15 mg/mL. Mel and PLA were stored at −20 °C, and 24 h before the experimental treatments, they were dissolved in bidistilled water at concentrations of 2.5 mg/mL and 5 mg/mL, respectively. For MNPs functionalization, Mel was dissolved in bidistilled water at a concentration of 2.5 mg/mL and stored at −80 °C until use. To obtain aqueous solutions for cell incubation, BV, Mel, PLA, MNPs, and MNPs-Mel were reconstituted in the culture medium.

### 2.2. Melittin-Functionalized Magnetic Nanoparticles

Fe_3_O_4_ magnetic MNPs were synthesized and characterized as recently described by us [25]. The citrate-coated MNPs were washed (3 × 15 min) and resuspended in distilled water. A total of 1 mL of this suspension containing 1 mg of MNPs was mixed with 1 mL of an aqueous Mel solution containing 112.13 (±5.12) μg Mel and ultrasonicated (10 min) and mechanically shaken (10 min). The MNPs-Mel were magnetically separated, and the remaining unattached Mel was measured by UV-Vis spectroscopy (with a T92+ UV-VIS Spectrophotometer, PG INSTRUMENTS, Leicestershire, UK) from the supernatant. The properties of MNPs-Mel were assessed as previously described [25]: their crystalline structure by X-ray powder diffraction, magnetic properties by vibrating sample magnetometry, the heating efficiency by magnetic hyperthermia (at a frequency of 355 kHz and amplitudes between 10 and 60 kA/m), and the Mel content by UV-Vis spectroscopy (in the UV spectrum). The shapes of MNPs were observed by transmission electron microscopy (TEM, with a JEOL JEM-100CX II, Jeol, Tokyo, Japan), and their sizes were manually measured (for 500 MNPs).

### 2.3. Cell Cultures

Two cell lines were used in this study: human immortalized skin keratinocytes (HaCaT cell line, Lot no: 300494-4517) obtained by Cell Line Service of the German Cancer Research Center (Heidelberg, Germany), and human melanoma cells (A375 cell line) from the European Collection of Authenticated Cell Cultures delivered by Sigma Aldrich (St. Louis, MO, USA). Both cell lines were cultured in standardized laboratory conditions (37 °C and an atmosphere of 5% CO_2_), with Dulbecco’s modified Eagle’s medium, 4.5 g glucose/L, supplemented with 10% fetal bovine serum, 2 mM L-glutamine, and 1% antibiotics.

### 2.4. Alamar Blue Viability Assay

To select the effective doses of the tested substances, their 50% inhibitory concentrations (IC_50_) were determined by the Alamar blue viability assay. This test was used to measure cell viability in the two lines incubated for 24 h with BV, Mel, MNPs, and MNPs-Mel at the concentrations listed in Table 1 and Table 2. The concentrations of Mel and PLA were equivalent to their content in the BV, while the concentrations of MNPs-Mel were calculated starting from the adsorbed Mel content.

Alamar blue assay is a nontoxic alternative to the MTT test, also used for assessing cellular viability. Alamar blue contains resazurin, a blue, cell-permeable dye that is weakly fluorescent. This molecule is redox-sensitive and undergoes color shifts in response to cellular metabolic changes. In its reduced form, resorufin is pink and has an intense fluorescence that increases with the number of living cells tested.

The HaCaT and A375 cells were seeded on 96-well plates (Delta Nunclon type) at a density of 10^4^ cells in 200 µL medium/well. After 24 h, when cells reached 60–70% subconfluency, the tested solutions of serial dilutions were added (BV, Mel, MNPs, and MNPs-Mel), and the cells were cultivated for an additional 24 h. At 24 h and 72 h post-treatment, the supernatant from the wells was replaced with 100 µL medium with 10% Alamar blue. After a 1 h incubation in the dark at 37 °C and in an atmosphere of 5% CO_2_, the plates were analyzed with a BioTek Synergy 2 microplate reader (Agilent BioTek, Winooski, VT, USA), using 560/590 nm (excitation/emission) filter settings.

Since Mel is the most relevant BV molecule for our study, the results of the viability assays allowed us to select the dose of 3 μg Mel/mL, as a low but effective one. To assess a potential dose–effect relation, a second dose of 6 µg/mL Mel was also selected. These doses are consistent with effective in vitro ranges (1–10 μg/mL) [16,25,34,35]. Starting from this point, the relevant (and equivalent) BV doses of 6 and 12 µg/mL were also established. Concerning the PLA testing, its cytotoxic effects were generated at much higher doses than those equivalent to the PLA concentration in BV. Therefore, this molecule was excluded from further tests.

### 2.5. Magnetic Hyperthermia

The HaCaT and A375 cells were cultured in triplicate in 6-well plates at a density of 2.5 × 10^5^/well (HaCaT) and 2.0 × 10^5^/well (A375) in 2.5 mL culture medium. After complete adherence of cells to the plates’ surface, the cells were incubated for 24 h with MNPs (50 and 100 µg/mL) or with MNPs-Mel (50 and 100 µg/mL, equivalent to 3 or 6 µg/mL Mel adsorbed). At the end of incubation time, the cells were trypsinized, resuspended in 1 mL fresh medium and subjected to a 30 min MH at 37 °C, according to the protocol recently described by us, in an Easy Heat 0224 system (Ambrell, Scottsville, NY, USA), at frequency of 355 kHz and amplitude of 25 kA/m, which are parameters established during preliminary tests [25]. The cells were cultured for an additional 24 h on fresh medium in 6-well plates and then detached from the plates and counted with an Eve^TM^ counter (NanoEntek, Seoul, Republic of Korea). Cells resuspended in 1 mL complete medium were submitted to an apoptosis assay. A second technical replicate was performed under identical conditions, and the cells were submitted to TEM analysis.

### 2.6. Annexin V/Propidium Iodide Assay

The cellular reactions to the various experimental treatments (two doses of BV, Mel, MNPs, and MNPs-Mel, with or without MH) were evaluated with the Dead Cell Apoptosis Kits with Annexin V for Flow Cytometry (Invitrogen, Thermo Fisher Scientific, Waltham, MA, USA), according to the kit protocol. The untreated cells were used as a negative control. The cells were washed with cold phosphate-buffered saline (PBS), centrifuged for 5 min at 500× *g*, and re-suspended in 100 µL annexin binding buffer. To this suspension, 5 µL Alexa Fluor™ 488 Annexin V (Component A) and 1 µL propidium iodide solution (100 µg/mL) (Component B) were added to each 100 µL of cell suspension. This step was followed by a 15 min incubation at room temperature. At the end, to each sample, another 500 µL of annexin binding buffer (Component C) was added. The cells were placed on ice until they were analyzed with a S3e Cell sorter (Bio-Rad Laboratories, Hercules, CA, USA).

### 2.7. Ultrastructural Analysis

The cells collected after the various incubation conditions and intervals were centrifuged for 7 min at 500× *g* and fixed in 1.5% osmium tetroxide (Sigma-Aldrich, St. Louis, MO, USA) solution in 0.15 M phosphate buffer (pH 7.4) for 1.5 h. The samples were dehydrated in an acetone series (50–100%, 30 min each) and infiltrated with EMBed-812 (Electron Microscopy Sciences, Hatfield, PA, USA) of increasing concentrations in acetone (30%, 50%, and 70%; 1 h each and 100% overnight). Sections of 60–70 nm cut with an Ultra 45◦ diamond knife (DiATOME AG, Nidau, Switzerland) on a Bromma 8800 ULTRATOME III ultramicrotome (LKB Produckter AB, Bromma, Sweden) were collected on Ø 3 mm 300 mesh copper grids (Agar Scientific Ltd., Stansted, UK) and contrasted for 12 min with 13% ethanol solution of uranyl acetate (Merck, Darmstadt, Germany), and for 4 min with 2.8% aqueous solution of lead citrate (Fluka AG, Buchs, Switzerland). Sections were examined with a JEOL JEM-100CX II (Jeol, Tokyo, Japan) transmission electron microscope, operating at 80 kV, equipped with a MegaView G3 camera running with Radius 2.1 software (both from Emsis, Münster, Germany).

### 2.8. H2AX Immunocytochemical Staining of A375 Cells

From a third technical replicate (as described in Section 2.5), cells derived from the hyperthermia experiments were reseeded on 8-well chamber slides and cultured for 24 h in complete culture medium. The next day, the cells were fixed with 4% paraformaldehyde for 15 min, then permeabilized with 0.02% Triton X100 for 15 min. Nonspecific antibody fixation was blocked with 10% bovine albumin for 15 min. Exposure to primary monoclonal antibody goat-anti-H2AX (Sigma-Aldrich) at a ×100 dilution was performed overnight at 4 °C, followed by incubation with secondary antibody anti-goat IgG FITC (Santa Cruz Biotechnologies, Dallas, TX, USA). Each step was followed by three washes with PBS. Finally, the nuclei were stained with a mounting medium containing DAPI (4′,6-diamidino-2-phenylindole) and fluorescence images were visualized with an Axiovert D1 microscope (Zeiss, Oberkochen, Germany) using filters 346 nm for DAPI and 488 nm for FITC staining. Images were captured with an AxioCam MRc CCD camera (Zeiss, Oberkochen, Germany).

### 2.9. Clonogenic Assay

To evaluate the ability of A375 cells to form colonies 24 h after applying hyperthermia, they were counted, seeded (100 cells/well) in a 24-well plate, and cultured for 7 days (in the same conditions as previously mentioned). The cells were then washed with PBS, fixed with methanol for 30 min, and stained with crystal violet for 5 min. The excess crystal violet was washed with distilled water, and the plates were left to dry. Colony counting was performed manually on recorded digital images. The results were calculated for plating efficiency = number of colonies counted/number of cells plated.

### 2.10. Experimental Treatments and Groups of Cells

Twenty-six groups of cells were analyzed in this study, 13 for either HaCaT or A375 cells, as presented in Table 3:

## 3. Results and Discussion

### 3.1. MNPs-Mel Characterization

TEM examination of MNPs-Mel revealed a relatively homogeneous population of mostly round MNPs (Figure 1a), with an average diameter of 45.37 ± 10.45 nm and 65% being in the range of 35–55 nm (Figure 1b).

The UV-Vis spectroscopy showed a concentration of Mel adsorbed on MNPs of 52.42 μg Mel/mg MNPs (corresponding to approximately 3 μg Mel/50 μg MNPs). Therefore, two doses of MNPs-Mel: 50 µg/mL and 100 µg/mL were also selected for the incubation experiments, which corresponded to 3 µg/mL and 6 µg/mL of adsorbed Mel, and were confirmed as effective by the viability assays. This way, we could compare the effects of similar doses of Mel, either free or adsorbed on the MNPs.

The magnetite nanoparticles used here contained both Fe^2+^ and Fe^3+^ ions arranged in an inverse spinel crystal structure. The magnetic properties of magnetite depend on crystal composition, size, shape, structural defects, surface spin organization, and interparticle interactions [36,37]. When Fe_3_O_4_ particles are sufficiently small and consist of a single magnetic domain, thermal fluctuations can randomize their magnetic moments in the absence of an external field. Such particles may exhibit superparamagnetic properties, becoming strongly magnetized under an applied magnetic field while showing minimal remanent magnetization after the field is removed. This behavior is advantageous for biomedical dispersions because it permits external magnetic manipulation while reducing permanent magnetic attraction and aggregation after field removal [36,37,38,39]. However, in the presence of an AMF, the MNPs increase the local temperature by several degrees [40], and tumor tissues are more susceptible to heat because of heterogeneous vascularization, hypoxia, and acidic conditions. Nevertheless, these characteristics vary among tumors and do not ensure selective damage. MNP distribution and local heat generation are often heterogeneous, and excessive heating can injure adjacent normal cells. Early clinical investigations in prostate cancer and recurrent glioblastoma established the feasibility of intratumoral iron-oxide MNPs administration followed by AMF exposure, while also demonstrating challenges related to particle distribution, thermal dosimetry, and treatment control [39,40]. The biological performance of Fe_3_O_4_ MNPs depends not only on their magnetic core but also on their hydrodynamic diameter, size distribution, morphology, surface charge, coating, colloidal stability, protein-corona formation, and tendency to aggregate. Bare magnetite surfaces may oxidize, interact nonspecifically with biomolecules, and aggregate in physiological media. Surface engineering is therefore commonly used to improve stability and provide functional groups for the attachment of peptides, drugs, antibodies, or other targeting molecules [29,36,37,38]. Additionally, the transported biological agent could be released from the MNPs inside the cells during MH exposure.

### 3.2. Cellular Viability

Cellular metabolic viability was evaluated using the Alamar Blue assay after 24 and 72 h of exposure to the investigated treatments (Figure 2). Fluorescence values were normalized to the corresponding untreated control, which was set at 100%, and the results were expressed as percentage viability. For each cell line and treatment, the effects of concentration and exposure time were analyzed using two-way repeated-measures ANOVA, with concentration and time included as experimental factors and measurements matched across rows according to the independent experimental runs. The analysis evaluated the main effects of concentration and time, as well as the concentration × time interaction. When a significant concentration × time interaction was identified, Šídák-adjusted multiple comparisons were used to compare the 24 h and 72 h responses at each concentration.

The significant concentration × time interactions indicate that neither whole BV nor Mel produced a uniform time-dependent effect across the complete concentration range. At higher concentrations, extending exposure to 72 h markedly enhanced the reduction in A375 metabolic viability. Bee venom concentrations of 12.5–250 µg/mL reduced mean viability from 39% at 24 h to 15% at 72 h. A similar pattern was observed for Mel, for which concentrations of 12.5–125 µg/mL reduced viability from 37% at 24 h to 16% at 72 h. These results suggest that, above a concentration threshold, the initial cellular injury was sustained or progressed during prolonged exposure. At lower concentrations, however, the response was different. A375 viability at 72 h approached or exceeded the matched control level at 6.25 µg/mL BV and 3.125 µg/mL Mel, despite an appreciable reduction at 24 h.

Because Alamar Blue measures cellular reducing activity rather than cell death directly, this apparent recovery may reflect survival and proliferation of a resistant cell population or adaptive changes in metabolism.

In A375 cells treated with MNPs-Mel, two-way repeated-measures ANOVA showed a significant concentration × time interaction ([F(9,20) = 99.7,\ *p* < 0.001]), indicating that the effect of exposure time varied across the tested concentrations. Significant main effects were also observed for concentration ([F(9,20) = 229,\ *p* < 0.001]) and time ([F(1,20) = 3868,\ *p* < 0.001]). Exposure time accounted for 53.6% of the total variation, concentration for 33.3%, and their interaction for 12.4%. Mean metabolic viability decreased from 83.9% at 24 h to 30.0% at 72 h, corresponding to a mean difference of 53.9 percentage points (95% CI: 52.1–55.7) (Figure 3).

In the present study, the 24 h viability curve crossed the 50% level between 3.125 and 6.25 µg/mL, which is broadly consistent with the reported IC_50_ [15]. Lim et al. also demonstrated concentration-dependent inhibition of A375SM melanoma-cell growth after 72 h, reporting IC_50_ values of 5.69 µg/mL for BV and 4.43 µg/mL for Mel [17]. The concentration threshold observed after 72 h in this study appeared somewhat higher, particularly for Mel, as viability decreased sharply between 6.25 and 12.5 µg/mL.

The membrane affinity of Mel to membranes results from its amino-acid sequence with a predominantly hydrophobic N-terminal region and a strongly basic C-terminal region. In aqueous solution, Mel is conformationally flexible, and its aggregation state depends on concentration, ionic strength, and pH. Upon interaction with lipid membranes, it preferentially adopts an amphipathic α-helical conformation in which hydrophobic and positively charged residues are distributed on opposite faces of the helix [16,41,42]. This structure enables Mel to bind efficiently to phospholipid bilayers through a combination of electrostatic and hydrophobic interactions. The initial interaction of Mel with a cell membrane generally involves adsorption parallel to the membrane surface. As the peptide-to-lipid ratio increases, Mel perturbs lipid packing, reduces bilayer thickness, modifies membrane curvature, and may reorient toward a transmembrane position. The resulting water permeability changes (measured by NMR by Bâlici et al. [43]) have been explained through several related models, including transient defects, toroidal pores, disordered peptide–lipid pores, and, at high concentrations, detergent-like membrane disruption [41,42,43,44,45,46]. The exact mechanism is influenced by peptide concentration, lipid composition, cholesterol content and membrane charge, as well as by the exposure time. Melittin should therefore not be viewed as having a single fixed membrane mechanism. Nevertheless, both treatments (with BV and Mel) displayed a similar threshold-like response, supporting the central contribution of Mel to BV cytotoxicity. At sufficiently high concentrations, the sustained reduction in metabolic viability may reflect a combination of apoptosis, inhibition of proliferation and direct membrane injury. Previous studies have shown that Mel induces caspase-dependent apoptosis in melanoma cells [17,18] while high peptide concentrations can also cause rapid membrane swelling, blebbing and lytic disruption [14]. Consequently, complementary Annexin V/PI and cell-recovery data are required to distinguish regulated apoptosis from extensive membrane damage in the most strongly affected treatment groups.

Phospholipase A_2_ is the second most abundant molecule of BV, also partially responsible for its general effects. Phospholipase A_2_ enzymes constitute a diverse superfamily that hydrolyses the ester bond at the *sn*-2 position of glycerophospholipids, producing a free fatty acid and a lysophospholipid [47,48]. When arachidonic acid is released from membrane phospholipids, it can serve as a precursor for eicosanoid synthesis. Lysophospholipids and free fatty acids can modify membrane curvature, permeability, intracellular signaling, and inflammatory responses. However, the biological consequences depend on the enzyme type, substrate composition, local concentration, and cellular environment. Hydrolysis by PLA can compromise membrane integrity through two related processes. First, it removes intact phospholipids from the bilayer. Second, it generates lysophospholipids and fatty acids whose molecular geometry differs from that of the original phospholipids. Accumulation of these products alters lipid packing and membrane properties, leading to vesiculation, rupture, or increased susceptibility to other membrane-active molecules. At high concentrations or under favorable conditions, these effects contribute to cytolysis and tissue injury.

However, in our experimental conditions, PLA produced comparatively limited effects on cellular metabolic viability. In A375 cells, two-way repeated-measures ANOVA identified a significant concentration × time interaction, together with significant effects of concentration and exposure time. In HaCaT cells, the concentration × time interaction was not significant, and no significant overall concentration effect was detected. The absence of both a concentration effect and a concentration × time interaction indicates that purified PLA did not produce a consistent dose-dependent reduction in HaCaT metabolic viability under the investigated conditions.

Taken together, these findings suggest that PLA alone was considerably less cytotoxic than BV or free Mel at the tested dose (equivalent to its concentration in the BV). Although statistically significant concentration-dependent differences were detected in A375 cells, the high overall viability values and the increase in mean viability at 72 h indicate that these differences represented relatively modest changes in cellular metabolic activity rather than progressive cytotoxicity. In HaCaT cells, the absence of a concentration effect further supports the limited direct activity of the isolated enzyme. Because Alamar Blue measures cellular reducing capacity, values approaching the untreated control after 72 h may reflect continued proliferation, metabolic adaptation, or recovery from an early and reversible effect. The limited cytotoxicity of isolated PLA is consistent with previous studies. Yaacoub et al. found that PLA alone did not significantly reduce HCT116 colon-cancer-cell viability at concentrations up to 50 µg/mL and reported an EC_50_ above 50 µg/mL. However, combining BV PLA with Mel markedly enhanced cytotoxicity, particularly when cells were exposed to Mel before PLA, supporting the hypothesis that Mel-mediated membrane perturbation facilitates access of the enzyme to phospholipid substrates [49]. Similarly, Putz et al. [50] demonstrated that the antitumor activity of BV secretory PLA was strongly enhanced by cooperation with phosphatidylinositol derivatives, indicating that its cytotoxic action depends substantially on membrane composition and the presence of cooperating membrane-active molecules [50].

In experimental models, relatively low doses of PLA have promoted the expansion or activity of Foxp3-positive regulatory T cells and reduced inflammatory responses. Such effects have been investigated in models of neuroinflammation, Parkinson-like disease, and chemically induced liver injury [51,52,53]. These observations emphasize that PLA is not uniformly pro-inflammatory or cytotoxic. Its biological action depends strongly on concentration, route of administration, cellular target, and the balance between direct membrane hydrolysis and secondary immune regulation [54,55].

Compared with Mel, the direct anticancer activity of purified PLA has been studied less extensively. Experimental work has nevertheless shown that PLA can interfere with tumor-cell membrane integrity and proliferation, especially when combined with phospholipid analogs or other membrane-active agents. In renal, breast, prostate, and transformed lung-cell cancer models, cooperative treatment involving PLA and phosphatidylinositol derivatives disrupted membranes, altered signaling, and generated tumor-cell material capable of activating dendritic cells [50]. These findings do not establish PLA as a universally selective anticancer agent, but they demonstrate that its phospholipid-hydrolyzing activity can be exploited under appropriately controlled conditions.

Overall, the data indicates that PLA is unlikely to be the principal component responsible for the marked cytotoxicity of bee venom in A375 cells when administered alone. Its biological importance may instead arise from its capacity to amplify membrane injury initiated by Mel. Melittin can disrupt lipid organization and expose phospholipid substrates, while PLA hydrolysis generates lysophospholipids and free fatty acids that further destabilize the membrane. Consequently, the comparatively weak effect of isolated PLA does not exclude an important contribution to the activity of whole BV in our experimental conditions; rather, it supports a cooperative mechanism in which PLA becomes substantially more effective in the presence of Mel or other membrane-active components. Therefore, based especially on the results collected from the viability tests, the effects of free PLA were not further tested in our study.

### 3.3. Apoptosis vs. Necrosis

In a previous study, we revealed the ability of MNPs-Mel to induce Caspase-9-dependent apoptosis in a Caco-2 cell line at 24 h and 72 h incubation [25]. In the current study, we used a different method for assessing more accurately the type of cell death. Thus, cell-death profiles were evaluated 24 h after treatment and MH exposure using Annexin V/PI staining. Cells were classified as viable, early apoptotic, late apoptotic, or necrotic. Total Annexin-positive apoptosis was calculated as the sum of the early- and late-apoptotic fractions, while necrosis was analyzed separately. Untreated A375 cells showed 94.55% viability, 0.20% early apoptosis, 4.85% late apoptosis, and 0.39% necrosis, corresponding to a background Annexin-positive apoptotic fraction of 5.05%. Untreated HaCaT cells showed 92.22% viability, 0.35% early apoptosis, 7.26% late apoptosis, and 0.17% necrosis, corresponding to 7.61% total Annexin-positive apoptosis. In several groups, this evaluation was not possible due to the extremely low number of remaining cells: HV2, AV2, AMNPs-Mel1-MH, and AMNPs-Mel2-MH. Figure 4 illustrates flow cytometry data recorded for the different experimental groups of HaCaT cells, and Figure 5 presents the flow cytometry results recorded for the groups of A375 cells.

In HaCaT cells, MNPs1 and MNPs2 without MH produced 14.18% and 15.13% total Annexin-positive apoptosis, respectively. The viable-cell fractions were 85.18% for MNPs1 and 84.46% for MNPs2 (Figure 6). Following MH, apoptosis increased markedly. MNPs1-MH produced 33.62% total apoptosis, consisting of 1.19% early and 32.43% late apoptosis, while viability decreased to 65.92%.

MNPs2-MH produced 25.75% total apoptosis, consisting of 0.73% early and 25.02% late apoptosis, with 72.89% viable cells. Therefore, in HaCaT cells, magnetic hyperthermia shifted the response from a mixed early- and late-apoptotic profile toward predominantly late apoptosis. MNPs-Mel without MH produced modest Annexin-positive responses in HaCaT cells. MNPs-Mel1 resulted in 87.55% viable cells and 11.80% total apoptosis, while MNPs-Mel2 resulted in 90.73% viable cells and 8.52% total apoptosis. The addition of MH substantially increased apoptosis. MNPs-Mel1-MH produced 25.56% total apoptosis, consisting of 1.80% early and 23.76% late apoptosis, and reduced viability to 73.99%. MNPs-Mel2-MH produced 29.45% total apoptosis, consisting of 1.73% early and 27.72% late apoptosis, and reduced viability to 70%. Compared with the corresponding treatment without MH, the Annexin-positive fraction increased by 13.76 percentage points for MNPs-Mel1 and by 20.93 percentage points for MNPs-Mel2. These findings indicate that MH promoted progression toward late apoptosis in HaCaT cells treated with MNPs-Mel.

In A375 melanoma cells, free Mel predominantly induced a late-apoptotic phenotype (Figure 6). The low dose of Mel, corresponding to 3 µg/mL Mel, resulted in 84.38% viable cells, 1.16% early apoptosis, 13.59% late apoptosis, and 1.39% necrosis, yielding 14.75% total Annexin-positive apoptosis. The high dose, corresponding to 6 µg/mL Mel, produced 85.64% viable cells, 0.48% early apoptosis, 11.58% late apoptosis, and 1.12% necrosis, corresponding to 12.06% total apoptosis. Thus, among the cells recovered for analysis, free Mel increased apoptosis relative to the untreated background, with most Annexin-positive cells already exhibiting loss of membrane integrity characteristic of late apoptosis.

Magnetic hyperthermia applied to unfunctionalized MNPs did not increase measurable Annexin positivity in A375 cells. MNPs1-MH resulted in 94.31% viable cells and 5.62% total apoptosis, whereas MNPs2-MH resulted in 94.79% viable cells and 5.15% total apoptosis. These values were close to the untreated apoptotic background. This finding indicates that, within the population remaining available for cytometric examination, MNPs-mediated hyperthermia alone did not produce a substantial apoptotic response 24 h after treatment. The most pronounced measurable apoptotic response in A375 cells was observed following treatment with MNPs-Mel1, containing the equivalent of 3 µg/mL Mel. This treatment reduced the viable-cell fraction to 71.64% and increased total Annexin-positive apoptosis to 28.30%, comprising 24.21% early and 4.09% late apoptosis. Necrosis remained limited to 0.05%. Relative to the untreated background, the total Annexin-positive fraction increased by 23.25 percentage points. The predominance of early apoptosis indicates that a substantial proportion of the recoverable melanoma cells had initiated apoptosis but had not yet progressed to advanced membrane permeabilization at the 24 h assessment.

MNPs-Mel2, containing the equivalent of 6 µg/mL Mel, produced 30.22% viable cells and 5.79% total apoptosis. The total number of cells was substantially decreased compared to the low concentration, and the apoptosis predominance was mildly shifted towards late apoptosis.

For both MNPs-Mel1-MH and MNPs-Mel2-MH, too few A375 cells remained for reliable Annexin V/PI acquisition. A comparison between cell types revealed a stronger measurable response to MNPs-Mel1 in A375 cells than in HaCaT cells. MNPs-Mel1 induced 28.30% apoptosis and reduced viability to 71.64% in A375 cells, whereas the same treatment produced 11.80% apoptosis and 87.55% viability in HaCaT cells. The Annexin-positive fraction was therefore 16.50 percentage points higher in A375 cells. This result suggests greater melanoma-cell sensitivity to the lower dose of MNPs-Mel.

At the second dose, MNPs-Mel2 produced 5.79% apoptosis in A375 cells and 8.52% in HaCaT cells. A direct numerical comparison between cell types was not possible for MNPs-Mel-MH because both hyperthermia-treated A375 groups contained too few cells for valid analysis, whereas sufficient HaCaT cells remained for quantification. Qualitatively, this difference suggests that the combined MNPs-Mel-MH treatment caused more extensive depletion of the recoverable A375 population.

Necrosis remained low in all quantifiable groups, ranging from 0 to 1.39% in A375 cells and from 0.17 to 1.36% in HaCaT cells. These findings indicate that the recovered and gated cell populations mainly exhibited apoptotic rather than primary necrotic characteristics. Nevertheless, the low necrotic fractions should not be interpreted as evidence that necrotic or lytic cell death was absent because the necrotic processes may have taken place in the time between treatment and cytometric reading.

Overall, the Annexin V/PI findings indicate that MNPs-Mel1 induced a marked early-apoptotic response in A375 melanoma cells, while the addition of MH resulted in such extensive cell depletion that the apoptotic phenotype could no longer be reliably quantified. In HaCaT cells, MH increased the apoptotic effects of both unfunctionalized and MNPs-Mel and promoted a predominantly late-apoptotic phenotype. The results therefore support a strong combined effect of Mel functionalization and magnetic hyperthermia, particularly in A375 cells.

The increase in Annexin-positive apoptosis induced by free Mel in A375 cells is consistent with previous reports describing melanoma cells as sensitive to the pro-apoptotic activity of this peptide. Lim et al. demonstrated that Mel markedly increased the combined early- and late-apoptotic population in A375SM melanoma cells after 24 h and activated caspases-3 and -9, supporting a caspase-dependent mechanism involving the mitochondrial apoptotic pathway [17]. The predominance of late apoptosis observed in the present study after treatment with free M1 and M2 may therefore indicate that, by the 24 h assessment, a substantial proportion of the responding cells had already progressed beyond the initial externalization of phosphatidylserine.

Melittin functionalization substantially altered the apoptotic profile compared with free peptide treatment. MNPs-Mel1 induced 28.30% of total apoptosis in A375 cells, compared with 14.75% following the equivalent 3 µg/mL concentration of free Mel. Moreover, free M1 produced predominantly late apoptosis, whereas MNPs-Mel1 produced 24.21% early apoptosis and only 4.09% late apoptosis. This difference suggests that attachment to MNPs modified the kinetics or cellular presentation of Mel rather than simply reproducing the effects of the freely dissolved peptide. A similar alteration in the mechanism of cell death was reported by Soman et al., who showed that nanoparticle-mediated delivery of Mel to C32 melanoma cells induced a concentration-dependent increase in early and late apoptosis, mitochondrial cytochrome c release, and comparatively little plasma-membrane lysis [56]. Even when more than 95% of the treated cells were undergoing death, the nanoparticle-delivered peptide produced no detectable primary necrosis, whereas free Mel caused substantially greater LDH release and membrane damage.

Other nanoparticle formulations have similarly shown that the carrier can modify both the availability and cytotoxicity of Mel. Hyaluronic-acid-coated Mel liposomes increased uptake and cytotoxicity in B16F10 melanoma cells compared with free Mel, while simultaneously reducing premature peptide leakage [57]. Qi et al. also demonstrated that a PEGylated graphene oxide–Fe_3_O_4_ magnetic nanocomposite could protect Mel and maintain its release for up to 72 h [58]. However, in HeLa cells, this system produced membrane pore formation and eventual cell lysis rather than a predominantly apoptotic response. The difference from the present findings illustrates that the mechanism of Mel-associated cell death depends not only on peptide concentration but also on nanoparticle composition.

The low measurable apoptosis after treatment with MNPs-Mel2 should be interpreted in this context. MNPs-Mel2 produced 5.79% Annexin-positive apoptosis and 94.04% viable cells among the A375 cells that remained available for acquisition, despite containing twice the Mel-equivalent dose of MNPs-Mel1. This non-linear response is unlikely to demonstrate that the higher dose was intrinsically less active. One possible explanation is that cells experiencing the most severe membrane damage died before the analysis was completed. A second, non-exclusive explanation is that increasing the amount of nanoparticle-associated Mel altered particle aggregation, surface accessibility, cellular uptake, or peptide release, so that the biologically available Mel did not increase proportionally with the nominal dose. The concentration-dependent framework proposed by Câmpian and Florea also indicates that Mel-functionalized nanomaterials may shift from regulated apoptotic injury at lower or intermediate exposure to rapid membrane disruption and cellular loss at higher exposure [15].

The inability to obtain sufficient A375 cells after MNPs-Mel1-MH and MNPs-Mel2-MH treatment provides additional evidence that Annexin V/PI percentages alone may underestimate the effect of highly cytotoxic conditions. This observation is consistent with the study of Câmpian et al., in which Mel-functionalized Fe_3_O_4_ MNPs produced antitumor effects in Caco-2 cells and MH enhanced the overall treatment response [25]. The present findings extend this principle to melanoma cells, as the combination of MNPs-Mel and MH caused a more extensive loss of recoverable A375 cells than MNPs-Mel alone.

A comparable enhancement of Mel nanotherapy by externally generated heat was reported by Farahavr et al. in A375 melanoma cells [59]. In that study, Mel was incorporated into anti-MUC18-functionalized gold nanocages, and near-infrared photothermal exposure increased the cytotoxic activity of the targeted formulation. Although photothermal therapy and MH rely on different energy sources, both results support the concept that localized heating can potentiate Mel-associated cellular injury.

The comparison with HaCaT cells suggests that the response to MNPs-Mel was cell-type dependent. At the first dose, MNPs-Mel1 induced 28.30% apoptosis in A375 cells but only 11.80% in HaCaT cells, while the corresponding viable fractions were 71.64% and 87.55%. This 16.50-percentage-point difference in Annexin-positive apoptosis supports greater melanoma-cell sensitivity to the lower dose of functionalized nanoparticles. Such preferential activity is compatible with previous Mel nanocarrier studies in which targeted or modified delivery enhanced accumulation and toxicity in melanoma cells while reducing immediate nonspecific peptide exposure [56,57].

Magnetic hyperthermia also produced a measurable late-apoptotic response in HaCaT cells. MNPs-Mel1-MH and MNPs-Mel2-MH resulted in 25.56% and 29.45% total apoptosis, respectively, with most Annexin-positive cells classified as late apoptotic. These findings indicate that the combined treatment was not exclusively damaging to melanoma cells and that MH can reduce the therapeutic separation between malignant and nonmalignant skin-derived cells. Nevertheless, sufficient HaCaT cells remained for acquisition after both combined treatments, whereas the corresponding A375 populations were too depleted to analyze. This qualitative difference is compatible with greater overall susceptibility of A375 cells, but it cannot be converted into a direct numerical comparison because the missing A375 values were treatment-related rather than random.

The response to unfunctionalized MNPs-MH was unexpectedly different between the two cell types. Apoptosis remained close to the untreated background in A375 cells, whereas MNPs1-MH and MNPs2-MH produced 33.62% and 25.75% apoptosis in HaCaT cells, predominantly at the late stage. Possible explanations include differences in nanoparticle uptake, intracellular iron processing, antioxidant capacity, membrane composition, and sensitivity to thermal or oxidative stress [15].

Taken together, comparison with previous studies supports the interpretation that MNPs functionalization modified the mode and kinetics of Mel action, with the lower MNPs-Mel dose favoring a marked early-apoptotic response in A375 cells. Magnetic hyperthermia further intensified the overall effect of MNPs-Mel. The results are consistent with earlier evidence that nanoparticle delivery can reduce immediate nonspecific Mel lysis, redirect its activity toward apoptosis, and permit potentiation by externally generated heat. At the same time, the response observed in A375 and HaCaT cells emphasizes that further optimization of Mel loading, MNPs concentration, and MH conditions is required to maximize the therapeutic window.

### 3.4. Ultrastructural Evaluation

When presenting and discussing the TEM results of our study, it is important to mention that the cells analyzed were the intact remaining ones that separated as pellets after the various experimental treatments (similar to the apoptosis test). Therefore, these TEM data correlate only partially with the other data. But this high-resolution method of visualization is essential to identify the fine, ultrastructural cellular and subcellular changes, which are fatal for many more sensitive cells, that eventually led to an important decrease in cell number in some of the analyzed groups. We recorded a large number of TEM images of our 26 experimental groups, but the main manuscript presents only the most relevant ultrastructural changes (mainly consequent to the incubation of cells with the high dose of tested agents), while the other ultrastructural results are organized as Supplementary Materials.

#### 3.4.1. Control HaCaT and A375 Cells

The control HaCaT cells (from the HC group) had large and polymorphic euchromatic nuclei, with prominent nucleoli (Figure 7a). Their cytoplasm contained a relatively reduced number of cell organelles: several oval/elongated mitochondria (of 0.5–0.8 µm), with well-defined cristae, a few primary lysosomes, rare and small autophagosomes (of 0.1–0.3 µm), electron–lucent vesicles and rare round lipid droplets (Figure 7a,b). Occasionally, profiles of endoplasmic reticulum and keratin filaments were also noted. The control A375 cells (AC group) contained large, euchromatic nuclei (Figure 7c–f) with prominent nucleoli (Figure 7c). In the cytoplasm, the numerous mitochondria showed distinctive aspects: some of them were elongated (0.5–1.1 µm), presenting an electron-dense matrix and visible transverse cristae (Figure 7c,d), while others had oval shapes (0.5–0.6 µm), with a granular matrix and fewer and smaller cristae (Figure 7c–f). Primary lysosomes were in low numbers (Figure 7c,e,f), the endoplasmic reticulum was scarce (Figure 7c–f), and the Golgi apparatus was rarely observed (Figure 7e). Additionally, several lipid droplets were observed in the cells of this group, with a homogeneous aspect and irregular shapes.

The size and structure of the nucleus and the aspect and number of cell organelles indicated sustained metabolic activity in both types of cells, especially in the A375 cells.

#### 3.4.2. Ultrastructural Effects of Cells Incubation with BV

The moderate ultrastructural effects of HaCaT cells incubated with the BV low dose (6 µg/mL)—group HV1—are presented in Figure S1. The high dose of BV (12 µg/mL) triggered important autophagosomal (increased in number and of 0.3–0.6 µm) and mitochondrial (disorganized cristae and rarefied matrix) reactions (Figure 8a,b), similar but more accentuated than those in the HV1 group. Additionally, a particular aspect noted was the presence of numerous lipid droplets with homogeneous aspect (Figure 8c), or presenting electron-dense, myelin-like content (Figure 8d).

In the A375 cells incubated with the low dose of BV (6 µg/mL)—group AV1—the prevalent effect was noted in mitochondria, which suffered deep ultrastructural alterations. Almost all mitochondria were polymorphic, with fewer or no visible cristae (Figure 9a–d), or they were ballooned, with either electron–lucent content (Figure 9a,b), or electron–lucent matrix (Figure 9a,c,d). Many others were very long (1.3–1.5 µm) and had aberrant shapes (Figure 9c), and certain ones showed cristae reorganization into circular structures (inset of Figure 9d). The secondary lysosomes showed a normal aspect (Figure 9b) or contained crystalline electron–lucent inclusions (inset of Figure 9b).

The high dose of BV (12 µg/mL) killed all the cells, and no cellular pellet was found in the tubes corresponding to the group AV2.

The presence of BV in the culture media surrounding the cells during 24 h of incubation resulted in ultrastructural changes in both types of cells. However, these changes depended on the cell line and on the dose. If the low dose of BV generated more autophagosomes in the HaCaT cells, suggesting the occurrence of intracellular damage, the high dose affected mostly mitochondria and lipids (apart from increased autophagy), thus impacting the cellular metabolism.

On the other hand, the A375 cells reacted to the low dose of BV mainly by mitochondrial degeneration, while the BV tested at high dose destroyed all the cells. This outcome explains the impossibility of obtaining flow cytometry data in the AV2 group, but is not in contradiction with the viability test results, where the incubation with the high BV dose resulted in a cellular viability of about 40%, which further decreased after 72 h. For the TEM evaluation, the cells were cultivated for an additional interval of 24 h post-incubation, when the BV molecules, already attached to the plasma membrane or having arrived inside the cells, continued to act. The differentiated response of the two cell lines to the BV incubation suggested a higher sensitivity of the tumor cells and a lower ability to defend themselves against the cytotoxic action of BV molecules. And because this distinct behavior was maintained during testing of all agents, we detailed here only the TEM results from the groups of A375 cells, the others being available in the Supplementary Materials.

#### 3.4.3. Ultrastructural Effects of Cells Incubation with Mel

The incubation of HaCaT cells with the two doses of Mel had similar results to the incubation with the low dose of BV. However, some particular aspects were noted: both doses decreased the density of lipid droplets, while the high dose also triggered an amplified autophagosomal reaction (increased number and sizes of 0.2–0.6 µm), with the presence of more secondary lysosomes; longer (1–1.2 µm) and polymorphic mitochondria were also noted (Figure S2). Such similarities were expected since the doses of Mel used were equivalent to those contained in the doses of BV.

The incubation of A375 cells with the low dose of Mel (3 µg/mL) especially produced mitochondrial degeneration and/or elongation (2–2.6 µm), stimulated the intracellular digestion, affected the endoplasmic reticulum, and generated polymorphous lipid droplets (Figure S3). The A375 cells exposed to the dose of 6 µg Mel/mL, in the group AM2, contained a high number of large (0.6–1.5 µm), polymorphic autophagosomes and many secondary lysosomes (Figure 10a–d). In some secondary lysosomes, electro-lucent crystalline inclusions were noted (Figure 10a), as in the case of the AV1 group. The mitochondria of these cells were also deeply affected: they were very long (1.5–3 µm), polymorphic, with fewer cristae (Figure 10b–d), or presenting electron–lucent content (Figure 10c,d). When found, the endoplasmic reticulum was expanded (Figure 10a). The lipid droplets were abundant, with normal or abnormal density and irregular shapes (Figure 10a–d). All these ultrastructural reactions, similar but of different amplitude, differentiate the recorded effects of the high doses of Mel from those of BV, a logical explanation being represented by the complex composition of the BV (as mentioned above). However, the reactions of mitochondria (exaggerated length, their polymorphism, and the reduced number of cristae) are indicative of the intense toxic stress produced by the presence of Mel (either free or from BV) [60,61].

#### 3.4.4. Ultrastructural Effects of Cells Incubation with MNPs

The high number of images recorded in the various groups of cells allowed us to hypothesize a cellular distribution of MNPs, important for understanding the ultrastructural effects generated by the experimental conditions, as well as all the other cellular reactions reported here. During the 24 h of incubation, the MNPs (either with or without Mel coating), once in contact with the plasma membrane, were internalized via receptor-independent endocytosis (Figure 11a). They were further accumulated inside endosomes with well-defined, continuous membranes (Figure 11b). From this point, the endosomes could follow two different paths. The first path implied the endosomes that accumulated great numbers of MNPs, which mechanically ruptured the membrane surrounding them; the number of points of rupture was proportional to the number of MNPs inside: from fewer points (3 in Figure 11c) to numerous points (Figure 11d). Following the complete destruction of the endosome, the MNPs initially remained in the same area of the cell, respecting the shape of the former vesicle (Figure 11e) and then dispersed further into the cytoplasm (Figure 11f). The second path involved the fusion of the endosome with a primary lysosome (Figure 11g). From this point, depending on the number of MNPs from the endosome, two converging scenarios could be described. In the case of endosomes with high numbers of MNPs (Figure 11h), their fusion with primary lysosomes led more quickly to the rupture of the membrane surrounding them (Figure 11i), with the release of the MNPs in the cytoplasm (Figure 11f). On the other hand, when endosomes contained a lower number of particles, the MNPs accumulated more slowly inside secondary lysosomes (Figure 11j). The final result, however, was similar: when a certain number of MNPs progressively arrived inside the lysosome (Figure 11k), the destruction of the lysosome occurred (Figure 11i), followed by the spreading of the MNPs in the cytoplasm (Figure 11f). The same mechanisms could be described for MNPs alone, while cell exposure to MH increased the speed of these processes due to the higher temperature of the MNPs during MH, as well as due to their movements triggered by the AMF.

In both tested lines, the cells showed an extraordinary ability to internalize high amounts of MNPs. A differentiated behavior was still noted in the two cell lines after 24 h of incubation with MNPs, and we report a higher ability of HaCaT cells to internalize large amounts of MNPs (Figure S4) than the A375 cells (Figure S5). The intracellular distribution of MNPs was also similar in the HMNP groups and AMNP groups, regardless of the tested dose. Thus, they were found inside large intact endosomes, proving that the endocytosis process was still ongoing at the moment of fixation. They were also found inside lysosomes and dispersed freely in the cytosol. However, the general ultrastructure of the cells was unchanged or suffered minor changes. Rare autophagosomes and more secondary lysosomes were noted in the cells of the HMNPs1 group and of the HMNPs2 group, respectively (Figure S4), and low-level reactions of the endoplasmic reticulum or mitochondria were observed in the groups AMNPs1 and AMNPs2 as well.

#### 3.4.5. Ultrastructural Effects of Incubation with MNPs and MH Exposure

The cells of groups HMNPs1-MH and HMNPs2-MH presented moderate mitochondrial alteration and lysosomal response (Figure S6). On the other hand, the A375 cells incubated with the doses of 50 µg MNPs/mL or 100 µg MNPs/mL and exposed to 30 min MH (groups AMNPs1-MH and AMNPs2-MH) showed mainly dose-dependent profound and almost generalized mitochondrial degeneration (aberrant shapes, length of 2–3.5 µm, diameters varying between 0.5 and 2 µm, and heterogenous matrix, with few or disorganized cristae) and lysosomal induction (Figure S7).

In our study, testing of cellular reaction consecutively to the MNP internalization and MH exposure served as a reference to discriminate between the actual effects of MNPs and the potential toxic effects produced by the temperature increase during the action of the AFM on MNPs-containing cells. The TEM data, showing no relevant changes produced in the HaCaT cells and predominantly mitochondrial and lysosomal alteration in the A375 cells, are consistent with the cell death assay, which indicated low impact of the experimental setting, implying MNPs and MH on the cell viability, regardless of the tested dose.

#### 3.4.6. Ultrastructural Effects of Incubation with MNPs-Mel

A reduced dose-dependent mitochondrial polymorphism was the main reaction noted in the cells of the HMNPs-Mel1 and HMNPs-Mel2 groups, along with a low degree of cellular vacuolation (Figure S8).

In the AMNPs-Mel1 group, only rare cells with internalized nanoparticles were found, and with a low number of MNPs-Mel, located inside endosomes or lysosomes. These cells showed mainly altered mitochondria, with degenerated cristae (Figure S9). Incubation with the high dose of MNPs-Mel (100 µg/mL) resulted in general alterations of the A375 cells, with only some of them still presenting an intact plasma membrane (Figure 12a). However, these cells contained fewer organelles, with expansion of the endoplasmic reticulum. The other cells of this group were profoundly altered by the experimental treatment, having rarefied cytoplasm, mitochondria with electron–lucent matrix, lipid droplets and autophagosomes still visible, and areas in which the plasma membrane appeared interrupted (Figure 12b).

The results reported in this section are consistent with the Alamar blue test that showed a relevant reduction in cell viability only in the AMNPs-Mel2 group, as well as with the apoptosis assay. They could also be explained by the high toxicity of Mel. The Mel molecules, even in low doses, were massively transported inside the cells at the surface of the internalized MNPs and had an increased possibility to interact at a higher rate with the organelles and DNA as compared to the simple incubation of cells with free Mel. Thus, the cells with more accumulated MNPs, as well as those with more free MNPs-Mel dispersed within the cytosol, were severely damaged and eliminated prior to fixation of cellular pellets. But it remains difficult to explain, in the absence of other methods of molecular investigation, the low-level of intracellular alterations, and implicitly the higher ability of the HaCaT cells to resist the Mel transported by the MNPs, despite the high amounts of MNPs arriving inside their cytoplasm. However, this finding is in line with the literature data showing lower sensitivity to Mel of normal cells [25,56].

#### 3.4.7. Ultrastructural Effects of Incubation with MNPs-Mel and MH Exposure

The HaCaT cells incubated with MNPs-Mel in doses of 50 µg/mL and 100 µg/mL and exposed to 30 min of MH (groups HMNPs-Mel1-MH and HMNPs-Mel2-MH) were the only cells from all H groups with relevant modified ultrastructure. They showed dose-dependent mitochondrial alteration along with lysosomal and autophagosomal reaction (Figure S10).

In the A375 cells incubated with the low dose of MNPs-Mel (50 µg/mL) and exposed to 30 min MH (group AMNPs-Mel1-MH), the MNPs-Mel were found either in contact with the cytosol (Figure 13a,c) following the disruption of endosomes, or inside rare lysosomes (Figure 13b,d). A reduced number of mitochondria were still oval, but altered, with denser, granular matrix and almost devoid of cristae (Figure 13a–c). However, most mitochondria observed were polymorphic, with extremely rarefied matrix and fewer or no visible cristae (Figure 13a–c). Rare autophagosomes were found in this group (Figure 13a–d), while many lysosomes showed abnormal ultrastructure, containing electron–lucent areas or disrupted membranes (Figure 13b–d). Lipid droplets were in low amounts and had normal aspects, but small electron–lucent vesicles were also present in some cells (Figure 13c). The incubation with the high dose of MNPs-Mel (100 µg/mL), followed by exposure to 30 min MH, killed all the cells, and no cellular pellet was found in the tubes corresponding to the group AMNPs-Mel2-MH.

The cells incubated with MNPs-Mel and exposed to MH showed the most accentuated ultrastructural alteration, again with a net distinction between the HaCaT cells and the A375 cells. The recorded reactions depended on dose, and despite the reduced number of viable cells remaining in the analyzed pellets, they were very extensive, and it remains possible that prolonged cultivation of cells post-MNPs-Mel incubation and exposure to MH would result in much lower viability, especially in the A375 cells.

### 3.5. Immunocytochemical Data

In our study, we assessed by immunocytochemistry the expression of the non-phosphorylated H2AX form in A375 cells following the various experimental treatments. Evaluation was performed at 48 h post-hyperthermia. Figure 14 shows that the cells surviving after the applied therapies display more frequent intranuclear green fluorescent signals (nuclear foci) in the case of AV1, AM1, AM2, AMNPs1, and AMNPs-Mel2 groups, and also in the samples with MH. The non-phosphorylated H2AX plays the role of a stable, baseline precursor to γ-H2AX, as a mechanism to protect and prepare the cells to respond and to manage the genomic stress. The observation of a sharp decrease indicates that the cell’s DNA damage response is active. Thus, unphosphorylated H2AX must be continuously present to form γ-H2AX foci upon DNA breakage, which is fundamentally linked to the cell’s repair capacity [62]. The half-life of *γ*H2AX foci after DNA damage is approximately 2–7  h in various cell types [63]. If the base H2AX pool is depleted, the cell cannot properly produce γ-H2AX during acute stress, drastically increasing susceptibility to further DNA damage [64]. H2AX, a member of the histone H2A family, contributes to DNA packaging and is a part of the nucleosome complex implicated in the cellular mechanisms responsible for maintaining genomic stability in response to DNA damage. Phosphorylation of H2AX (γH2AX) enables the repair of DNA double-strand breaks [65]. Various injuries such as chemical mutagens, replication inhibitors, ultraviolet light, ionizing radiation, and oncogenic transformation lead to DNA double-strand breaks (DSB). Phosphorylation of multiple substrates occurs after PI3-like kinases ATM, ATR, and DNA-PK activation. Histone H2AX becomes phosphorylated at serine 139 in the immediate vicinity of the break. γH2AX rapidly accumulates over the sites of DSB and can be detected in immunocytochemistry as discrete nuclear foci [66]. When cells were exposed to BV and Mel, an increase in γ-H2AX expression was found. Melittin initiates several physiological and molecular responses that finally trigger DNA damage: it induces oxidative stress with the generation of intracellular reactive oxygen species, leading finally to oxidative DNA damage [67]. In non-small cell lung cancer, Mel has been shown to act as a sensitizer by impairing the cell’s ability to repair DNA double-strand breaks. This causes, in turn, an increase in γ-H2AX [68]. The accumulation of DNA damage triggers intrinsic apoptotic pathways, characterized by the upregulation of pro-apoptotic proteins (like Bax) and down-regulation of anti-apoptotic proteins (like Bcl-2), eventually leading to cell death [24].

### 3.6. Clonogenic Assay

In Figure 15, the clonogenic potential of A375 cells results are representatives of three biological experiments. The dose of 6 µg BV/mL completely inhibited the clonogenic capacity of melanoma cells. Melittin and MNPs-Mel inhibited colony formation with a plating efficiency <50% compared with controls. Hyperthermia applied to cells treated with MNPs-Mel1 and MNPs-Mel2 enhanced this inhibition and reduced the plating efficiency drastically. Two-tailed *t*-tests performed between the treated samples revealed statistical differences only for the groups AM2 vs. AMNPs-Mel2 (*p* < 0.05) and AM2 vs. MNPs-Mel2-MH (*p* < 0.0.01). Similar findings with significant inhibition of the colony-forming ability were reported by Lim HN et al. (2019) after the melanoma cells B16F10, A375SM and SK-MEL-28 treatment with venom (2.5 µg/mL and 5 µg/mL) and Mel (2.5 µg/mL and 5 µg/mL) [17].

The novelty of this study lies in its dose-matched comparison of free Mel and Fe_3_O_4_-associated Mel, with and without MH, in paired malignant and nonmalignant skin-derived cell models. Rather than evaluating cytotoxicity alone, we integrated metabolic activity, absolute cell recovery, Annexin V/PI death phenotype, clonogenic survival, and ultrastructural observations to determine whether nanoparticle association changes the magnitude, kinetics, and apparent cell-type selectivity of the Mel response. BV and PLA_2_ were included as composition-based benchmarks and as a preliminary dose-selection step.

This study has a few limitations. First of all, we could not clearly identify the causality regarding the differences in sensitivity of the A375 cells to the treatments and to the increased apoptotic cell death in HaCaT cells. On the other hand, we could not establish with TEM whether there was a correlation between the tested dose and the amount of internalized MNPs, with more adequate quantitative methods being required to assess this important parameter.

## 4. Conclusions

Metabolic assessment clearly demonstrated dose × time-dependent effects, and TEM further confirmed the former. Under the conditions tested, purified PLA produced smaller changes in metabolic activity than BV or free Mel at composition-matched concentrations, supporting Mel as the principal cytotoxic BV component in this model. At the lower dose, MNP-associated Mel was linked to a larger Annexin-positive fraction and a predominantly early-apoptotic profile in A375 cells compared with HaCaT cells and dose-matched free Mel. MH further depleted the recoverable A375 population, but it also increased predominantly late apoptosis in HaCaT cells, indicating that greater treatment intensity may reduce cell-type selectivity. TEM supported intracellular nanoparticle localization and treatment-associated mitochondrial and lysosomal alterations, although it sampled only surviving pelletable cells. These in vitro findings justify further evaluation using complete thermal dosimetry, independently replicated dose–response analyses, normal-cell and hemolysis safety testing, and in vivo biodistribution and efficacy models; they do not yet establish tumor targeting or clinical effectiveness.

## Figures and Tables

**Figure 1:**
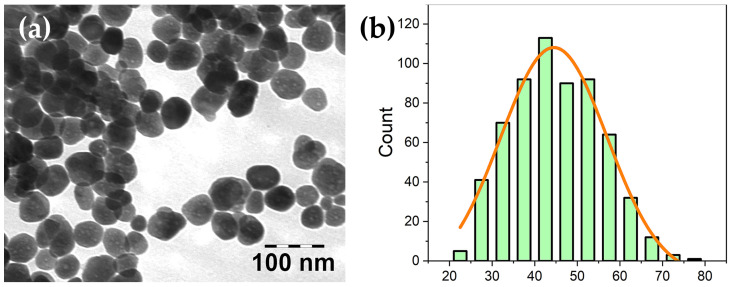
MNPs-Mel: (**a**) TEM image of MNPs-Mel. (**b**) Size distribution of MNPs-Mel fitted to a normal distribution. On the horizontal axis, the values are given in nm (n = 500).

**Figure 2:**
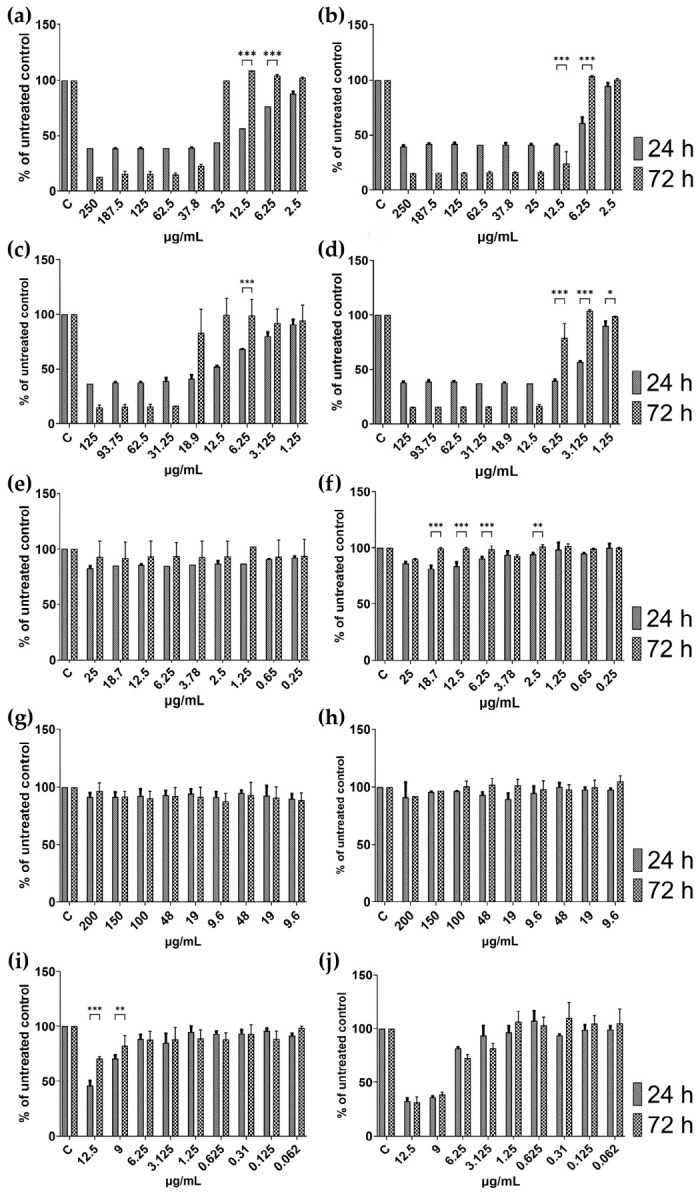
Metabolic activity of HaCaT cells (left panels) and A375 cells (right panels) after treatment with: (**a**,**b**) BV, (**c**,**d**) Mel, (**e**,**f**) PLA_2_, (**g**,**h**) MNPs, or (**i**,**j**) MNPs–Mel. (**i**,**j**): the concentrations tested are equivalent to the actual content of Mel adsorbed on the MNPs. Fluorescence was normalized to the corresponding time-matched untreated control (100%—always first column C) (n = 3); * *p* < 0.05, ** *p* < 0.01, *** *p* < 0.001.

**Figure 3:**
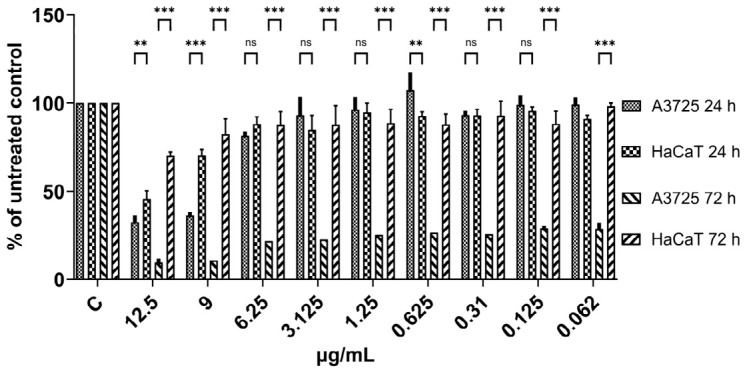
Metabolic activity of HaCaT and A375 cells after treatment with MNPs–Mel at 24 and 72 h, respectively. The concentrations tested are equivalent to the actual content of Mel adsorbed on the MNPs. Fluorescence was normalized to the corresponding time-matched untreated control (100%—first column C) (n = 3); ** *p* < 0.01, *** *p* < 0.001; ns not significant.

**Figure 4:**
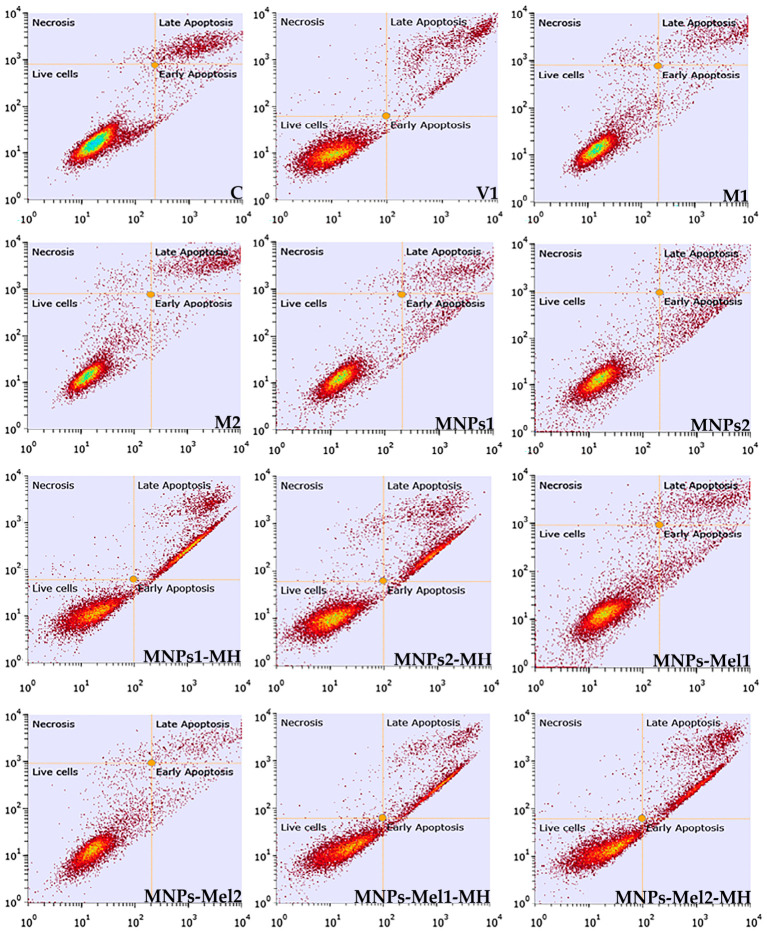
Flow cytometry data recorded for the different experimental groups of HaCaT cells.

**Figure 5:**
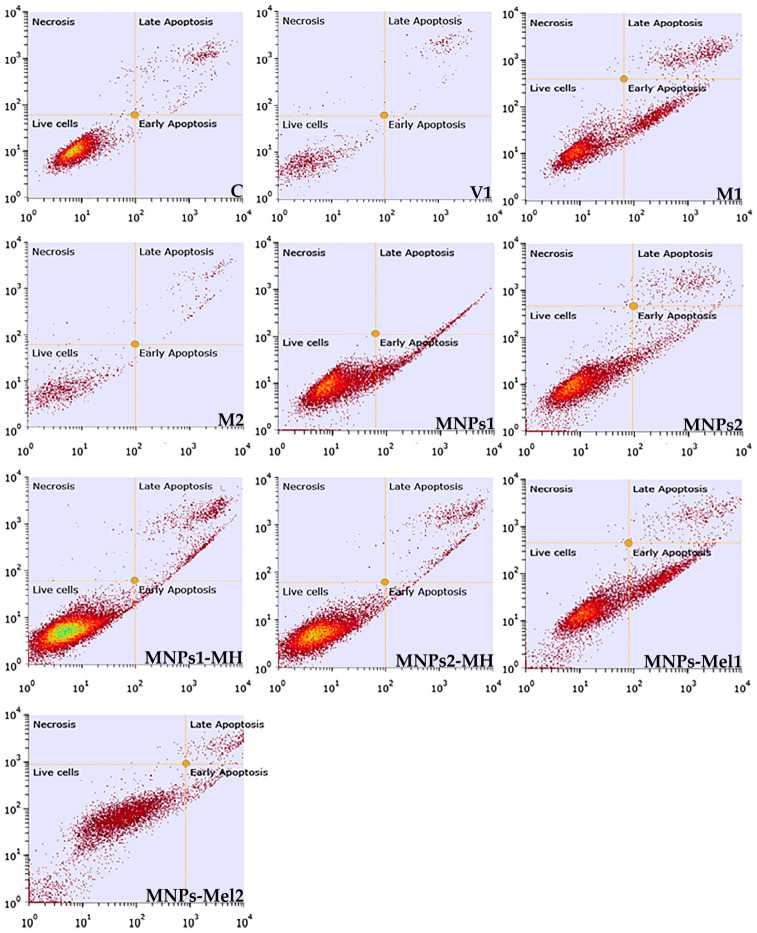
Flow cytometry data recorded for the different experimental groups of A375 cells.

**Figure 6:**
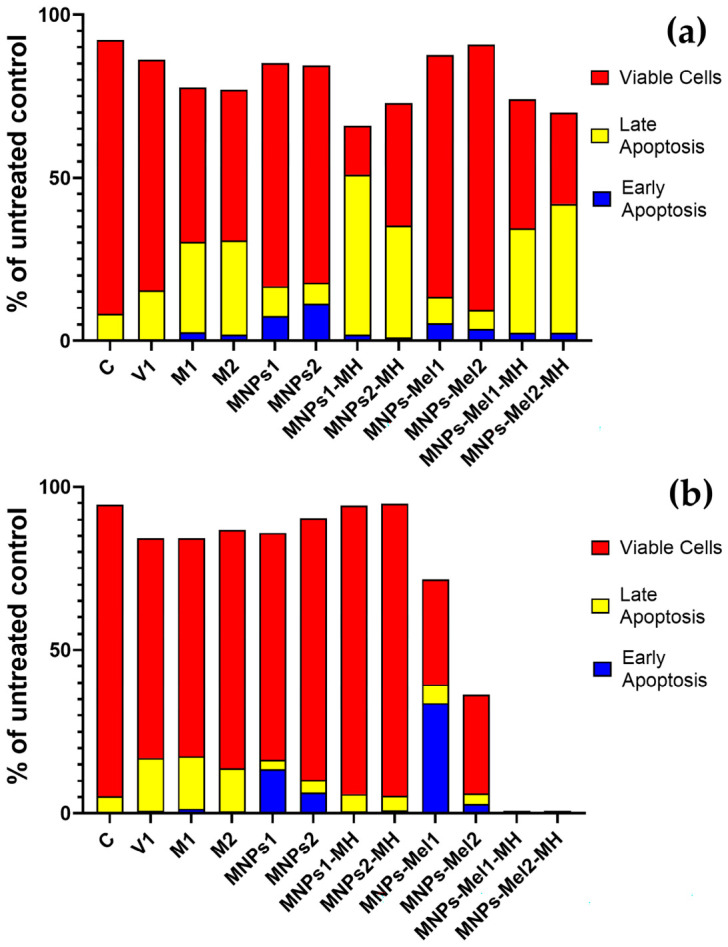
Annexin V/PI flow cytometry analysis of HaCaT (**a**) and A375 (**b**) cells, underlining the early and late apoptosis expressed as percentage of viable cells.

**Figure 7:**
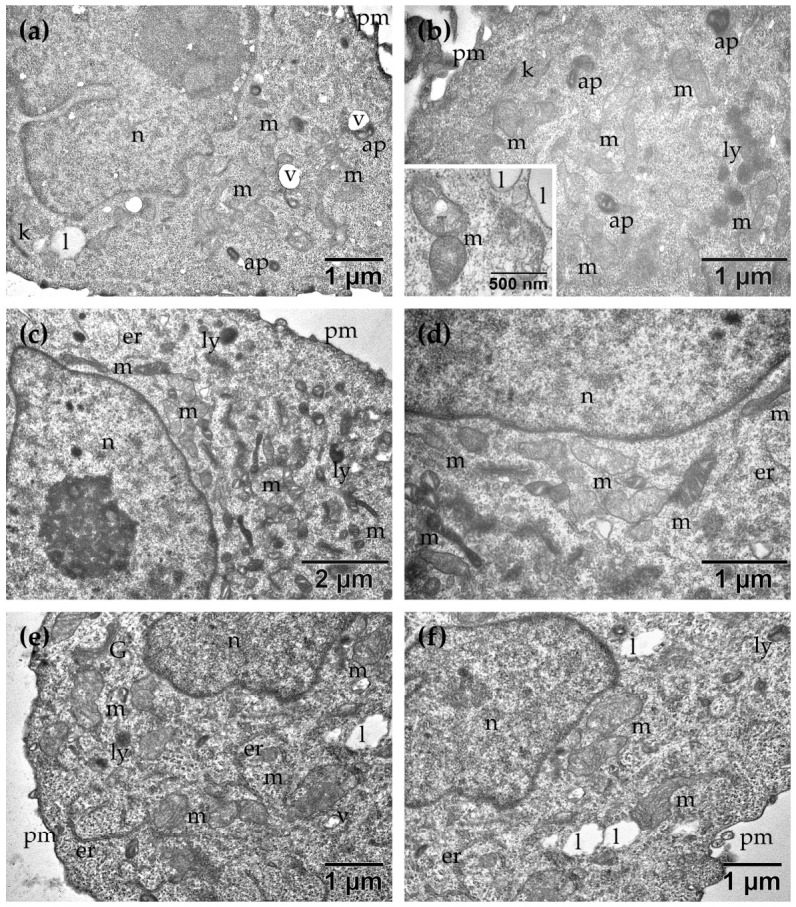
TEM images of cells in the HC (**a**,**b**) and AC (**c**–**f**) groups. ap: autophagosome; er: endoplasmic reticulum; G: Golgi apparatus; k: keratin filaments; l: lipid droplets; ly: lysosome; m: mitochondria; n: nucleus; pm: plasma membrane; v: vesicle.

**Figure 8:**
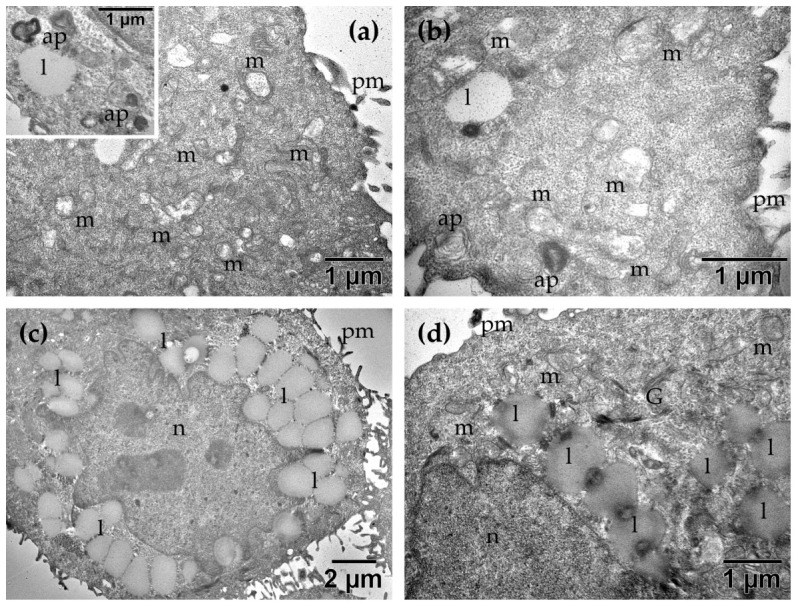
(**a**–**d**) TEM images of cells in the HV2 group. ap: autophagosome; G: Golgi apparatus; l: lipid droplets; m: mitochondria; n: nucleus; pm: plasma membrane.

**Figure 9:**
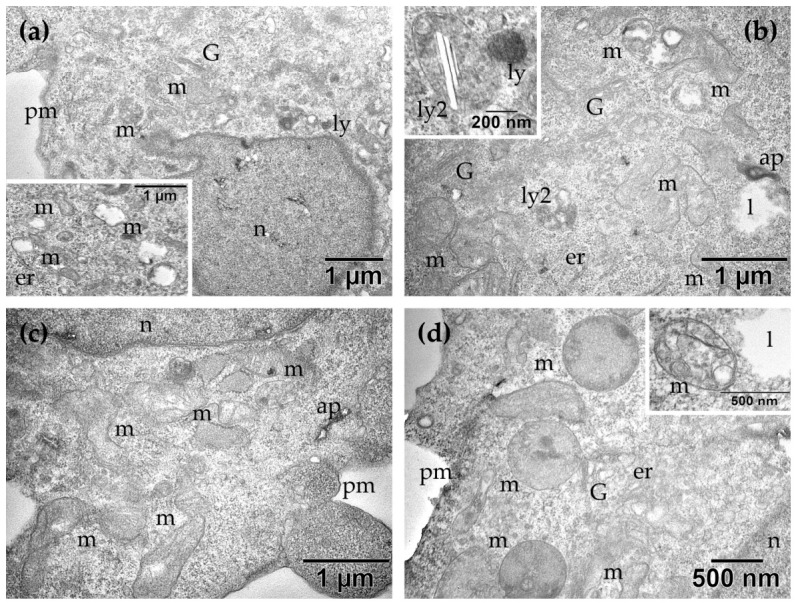
(**a**–**d**) TEM images of cells in the AV1 group. ap: autophagosome; er: endoplasmic reticulum; G: Golgi apparatus; l: lipid droplets; ly: lysosome; ly2: secondary lysosome; m: mitochondria; n: nucleus; pm: plasma membrane.

**Figure 10:**
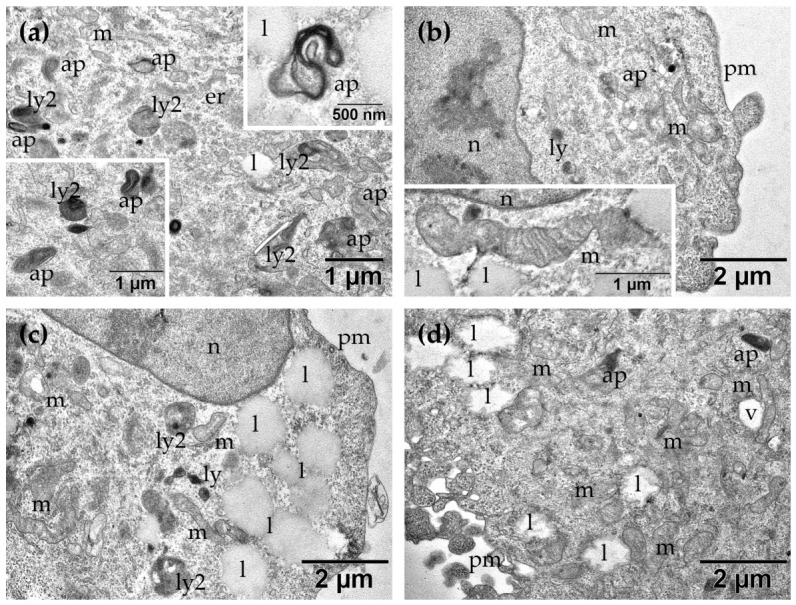
(**a**–**d**) TEM images of cells in the AM2 group. ap: autophagosome; er: endoplasmic reticulum; l: lipid droplets; ly: lysosome; ly2: secondary lysosome; m: mitochondria; n: nucleus; pm: plasma membrane; v: vesicle.

**Figure 11:**
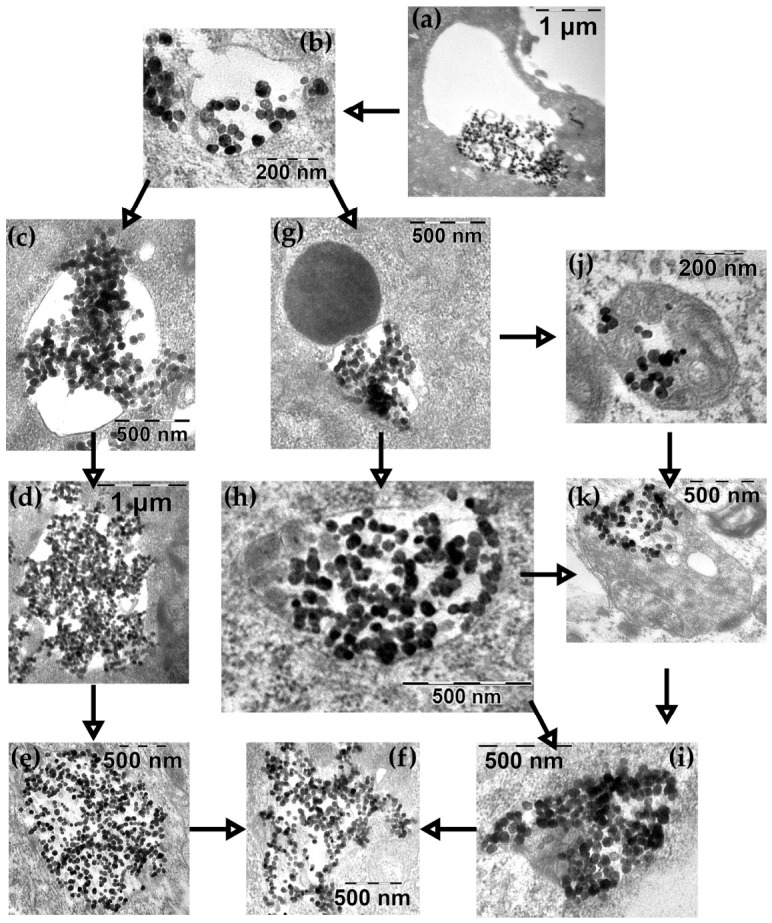
(**a**–**k**) Hypothesized cellular distribution of MNPs (see explanations in text).

**Figure 12:**
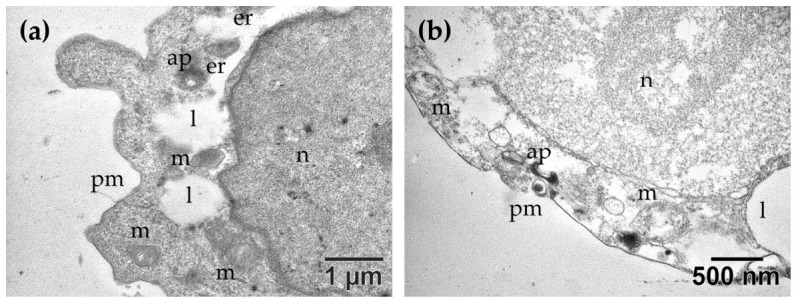
(**a**,**b**) TEM images of extremely altered cells in the AMNPs-Mel2 group. ap: autophagosome; er: endoplasmic reticulum; l: lipid droplet; m: mitochondria; n: nucleus; pm: plasma membrane.

**Figure 13:**
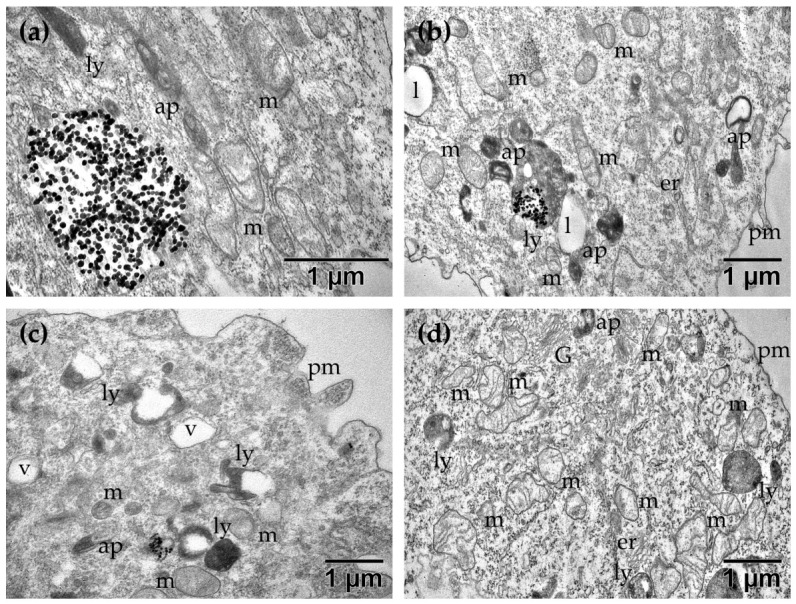
(**a**–**d**) TEM images of cells in the AMNPs-Mel1-MH group. ap: autophagosome; er: endoplasmic reticulum; G: Golgi apparatus; l: lipid droplet; ly: lysosome; m: mitochondria; pm: plasma membrane; v: vesicle.

**Figure 14:**
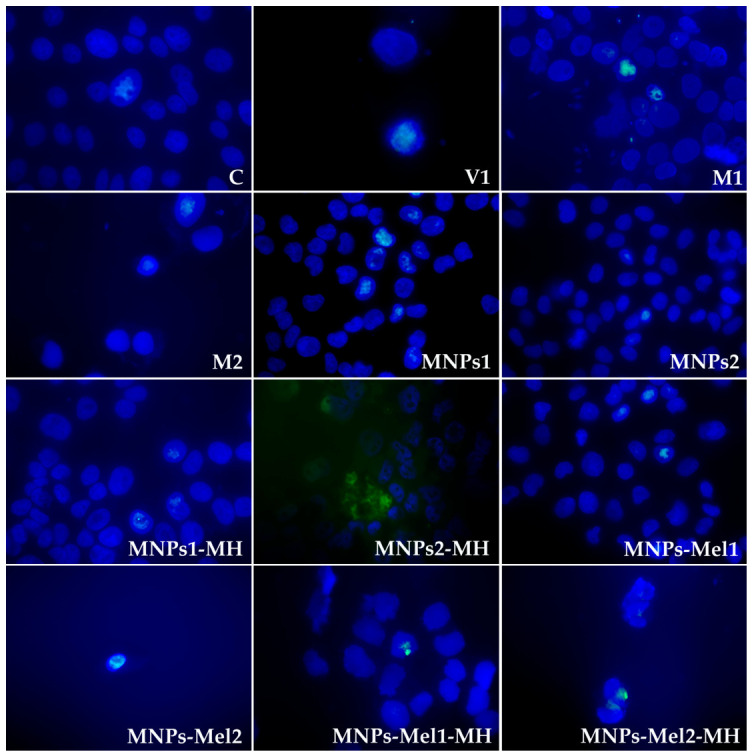
Merged fluorescence images of A375 cells stained with anti-H2AX antibody FITC and DAPI as nuclei counterstain (objective ×100).

**Figure 15:**
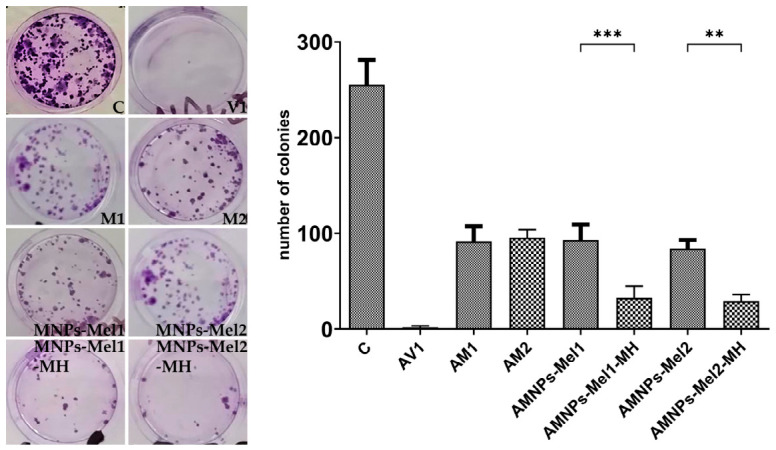
Clonogenic potential of various groups of A375 cells in response to the experimental treatments. ** *p* < 0.01, *** *p* < 0.001.

**Table 1:** Doses of BV, Mel and PLA tested in the viability assay.

Tested Substance	Ctrl	10	5	2.5	Stock × 10 15 µL	10 µL	5 µL	2.5 µL	1 µL
BV (µg/mL)	0	250	125	62.5	37.8	25	12.5	6.25	2.5
Mel (µg/mL)	0	125	62.5	31.25	18.9	12.5	6.25	3.125	1.25
PLA (µg/mL)	0	25	12.5	6.25	3.78	2.5	1.25	0.65	0.25

**Table 2:** Doses of MNPs and MNPs-Mel tested in the viability assay.

Tested Substance	1	2	3	4	5	6	7	8	9
MNPs µL	40	30	20	9.6	3.8	1.92	9.6	3.8	1.92
MNPs (µg/mL)	200	150	100	48	19	9.6	48	19	9.6
Mel eq to MNPs-Mel (µg/mL)	12.5	9	6.25	3.125	1.25	0.625	0.31	0.125	0.062

**Table 3:** Experimental treatments and groups of cells.

Group No.	Cell Line	Agent	Dose	MH	Group Code
1	HaCaT cells	-	-	-	HC
2	HaCaT cells	BV	6 µg/mL	-	HV1
3	HaCaT cells	BV	12 µg/mL	-	HV2
4	HaCaT cells	Mel (free)	3 µg/mL	-	HM1
5	HaCaT cells	Mel (free)	6 µg/mL	-	HM2
6	HaCaT cells	MNPs	50 µg/mL	-	HMNPs1
7	HaCaT cells	MNPs	100 µg/mL	-	HMNPs2
8	HaCaT cells	MNPs	50 µg/mL	30 min at 25 kA/m	HMNPs1-MH
9	HaCaT cells	MNPs	100 µg/mL	30 min at 25 kA/m	HMNPs2-MH
10	HaCaT cells	MNPs-Mel	50 µg/mL	-	HMNPs-Mel1
11	HaCaT cells	MNPs-Mel	100 µg/mL	-	HMNPs-Mel2
12	HaCaT cells	MNPs-Mel	50 µg/mL	30 min at 25 kA/m	HMNPs-Mel1-MH
13	HaCaT cells	MNPs-Mel	100 µg/mL	30 min at 25 kA/m	HMNPs-Mel2-MH
14	A375 cells	-	-	-	AC
15	A375 cells	BV	6 µg/mL	-	AV1
16	A375 cells	BV	12 µg/mL	-	AV2
17	A375 cells	Mel (free)	3 µg/mL	-	AM1
18	A375 cells	Mel (free)	6 µg/mL	-	AM2
19	A375 cells	MNPs	50 µg/mL	-	AMNPs1
20	A375 cells	MNPs	100 µg/mL	-	AMNPs2
21	A375 cells	MNPs	50 µg/mL	30 min at 25 kA/m	AMNPs1-MH
22	A375 cells	MNPs	100 µg/mL	30 min at 25 kA/m	AMNPs2-MH
23	A375 cells	MNPs-Mel	50 µg/mL	-	AMNPs-Mel1
24	A375 cells	MNPs-Mel	100 µg/mL	-	AMNPs-Mel2
25	A375 cells	MNPs-Mel	50 µg/mL	30 min at 25 kA/m	AMNPs-Mel1-MH
26	A375 cells	MNPs-Mel	100 µg/mL	30 min at 25 kA/m	AMNPs-Mel2-MH

## Data Availability

The original contributions presented in this study are included in the article/Supplementary Materials. Further inquiries can be directed to the corresponding author.

## Supplementary Materials

The following supporting information can be downloaded at https://www.mdpi.com/article/10.3390/ma19153260/s1; Figure S1: TEM images of cells in the HV1 group; Figure S2: TEM images of cells in the HM1 (a–d) and HM2 (e–h) groups; Figure S3: TEM images of cells in the AM1 group; Figure S4: TEM images of the HMNPs1 (a–d) and HMNPs2 (e–h) groups; Figure S5: TEM images of the AMNPs1 (a–d) and AMNPs2 (e–h) groups; Figure S6: TEM images of the HMNPs1-MH (a-d) and HMNPs2-MH (e–h) groups; Figure S7: TEM images of the AMNPs1-MH (a–d) and AMNPs2-MH (e–h) groups; Figure S8: TEM images of the HMNPs-Mel1 (a–d) and HMNPs-Mel2 (e–h) groups; Figure S9: TEM images of cells in the AMNPs-Mel1 group; Figure S10: TEM images of cells in the HMNPs-Mel1-MH (a–d) and HMNPs-Mel2-MH (e–h) groups.

## Abbreviations

The following abbreviations are used in this manuscript:

AC	Untreated A375 cells
AM1	A375 cells incubated with 3 µg/mL melittin
AM2	A375 cells incubated with 6 µg/mL melittin
AMF	Alternating magnetic field
AMNPs1	A375 cells incubated with 50 µg/mL magnetic nanoparticles
AMNPs2	A375 cells incubated with 100 µg/mL magnetic nanoparticles
AMNPs-Mel1	A375 cells incubated with 50 µg/mL melittin-functionalized magnetic nanoparticles
AMNPs-Mel2	A375 cells incubated with 100 µg/mL melittin-functionalized magnetic nanoparticles
AMNPs-Mel1-MH	A375 cells incubated with 50 µg/mL melittin-functionalized magnetic nanoparticles exposed to magnetic hyperthermia
AMNPs-Mel2-MH	A375 cells incubated with 100 µg/mL melittin-functionalized magnetic nanoparticles exposed to magnetic hyperthermia
AMNPs1-MH	A375 cells incubated with 50 µg/mL magnetic nanoparticles exposed to magnetic hyperthermia
AMNPs2-MH	A375 cells incubated with 100 µg/mL magnetic nanoparticles exposed to magnetic hyperthermia
AV1	A375 cells incubated with BV at a dose of 6 µg/mL
AV2	A375 cells incubated with BV at a dose of 12 µg/mL
BV	Bee venom
DMSO	dimethyl sulfoxide
HC	Untreated HaCaT cells
HM1	HaCaT cells incubated with 3 µg/mL melittin
HM2	HaCaT cells incubated with 6 µg/mL melittin
HMNPs1	HaCaT cells incubated with 50 µg/mL magnetic nanoparticles
HMNPs2	HaCaT cells incubated with 100 µg/mL magnetic nanoparticles
HMNPs-Mel1	HaCaT cells incubated with 50 µg/mL melittin-functionalized magnetic nanoparticles
HMNPs-Mel2	HaCaT cells incubated with 100 µg/mL melittin-functionalized magnetic nanoparticles
HMNPs-Mel1-MH	HaCaT cells incubated with 50 µg/mL melittin-functionalized magnetic nanoparticles exposed to magnetic hyperthermia
HMNPs-Mel2-MH	HaCaT cells incubated with 100 µg/mL melittin-functionalized magnetic nanoparticles exposed to magnetic hyperthermia
HMNPs1-MH	HaCaT cells incubated with 50 µg/mL magnetic nanoparticles exposed to magnetic hyperthermia
HMNPs2-MH	HaCaT cells incubated with 100 µg/mL magnetic nanoparticles exposed to magnetic hyperthermia
HV1	HaCaT cells incubated with BV at a dose of 6 µg/mL
HV2	HaCaT cells incubated with BV at a dose of 12 µg/mL
Mel	Melittin
MH	Magnetic hyperthermia
MNPs	Magnetic nanoparticles
MNPs-Mel	Melittin-functionalized magnetic nanoparticles
PLA	Phospholipase A_2_
TEM	Transmission electron microscopy
