# Supplementary material for: Mechanisms of In Vitro Cytotoxicity of Honeybee Venom Components and Melittin-Functionalized Fe3O4 Nanoparticles on HaCaT Keratinocytes and A375 Melanoma Cells
Source: Materials (Basel). 2026 Aug 1;19(15):3260. doi: 10.3390/ma19153260 (PMC13467246; doi:10.3390/ma19153260)
Supplement: Supplementary file 1 [file materials-19-03260-s001.zip › materials-4440867-supplementary.pdf]

materials

MDPI

Article - Supplementary Materials

Mechanisms of In Vitro Cytotoxicity of Honeybee Venom Components and Melittin-Functionalized Fe_3O_4 Nanoparticles on HaCaT Keratinocytes and A375 Melanoma Cells

Alex Câmpian [1], Olga Soriţău [2], Mara Muntean [1], Cristian Iacoviţă [3], Oana Baldasici [4], Otilia Andercou [2,*] and Adrian Florea [1]

[1] Department of Cell and Molecular Biology, Faculty of Medicine, "Iuliu Haţieganu" University of Medicine and Pharmacy, 6 Louis Pasteur St., 400349 Cluj-Napoca, Romania; alexcampian@yahoo.com (A.C.); muntean.mara@elearn.umfcluj.ro (M.M.); aflorea@umfcluj.ro (A.F.)

[2] Radiobiology and Tumor Biology Laboratory, The Oncology Institute "Prof. Dr. I. Chiricută", 34–36 Republicii St., 400015 Cluj-Napoca, Romania; olgasoritau@yahoo.com

[3] Department of Pharmaceutical Physics-Biophysics, Faculty of Pharmacy, "Iuliu Haţieganu" University of Medicine and Pharmacy, 6 Louis Pasteur St., 400349 Cluj-Napoca, Romania; cristian.iacovita@umfcluj.ro

[4] Department of Genetics, Genomics and Experimental Pathology, The Oncology Institute "Prof. Dr. Ion Chiricuţă", 34–36 Republicii St., 400015 Cluj-Napoca, Romania; oana_baldasici@yahoo.ro

* Correspondence: otiliabarbos2006@yahoo.com

1. Ultrastructural Effects of HaCaT Cells Incubation with the low dose of BV

The incubation of HaCaT cells with the dose of 6 µg BV/mL—group HV1—resulted in an increased number of autophagosomes (Figure S1a,b,d), also of larger sizes, and the presence of thicker keratin bundles (Figure S1a). Some mitochondria preserved a normal structure (Figure S1a,b), but most of them were ballooned, with an electron–lucent matrix (Figure S1a,c,d). Certain mitochondria suffered deep ultrastructural alterations, with re`organization of their cristae into circular structures (Figure S1d).

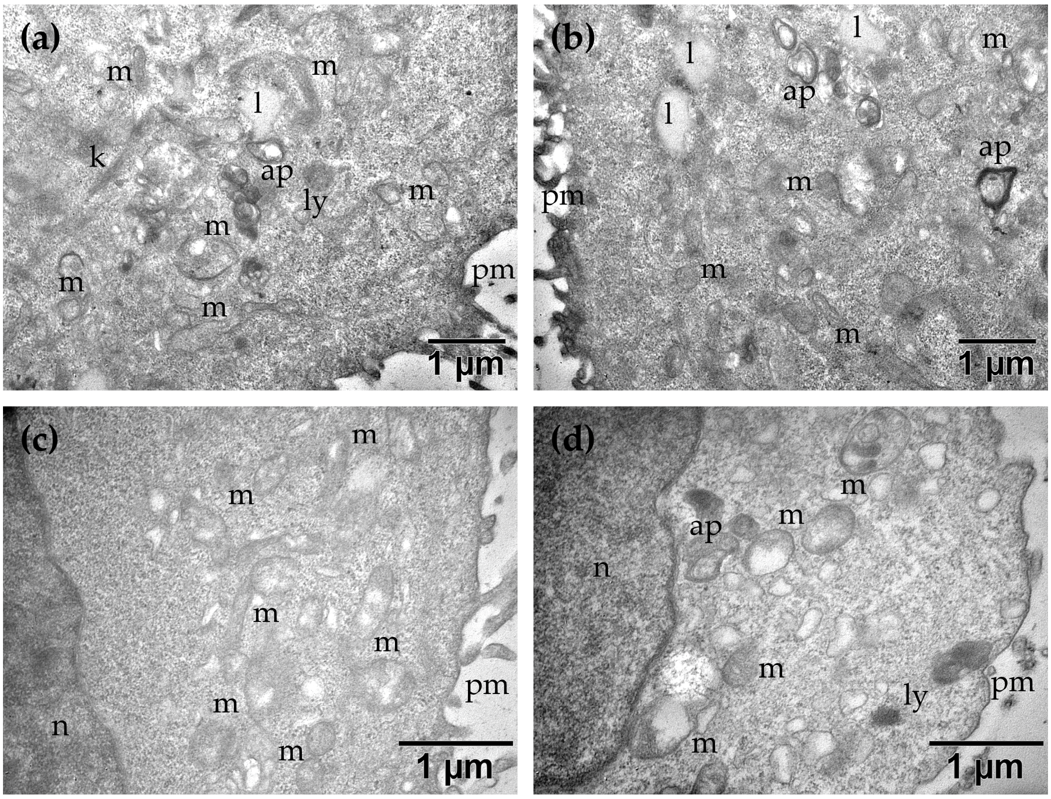

Figure S1. TEM images of cells in the HV1 group**.** ap: autophagosome; k: keratin filaments; l: lipid droplets; ly: lysosome; m: mitochondria; n: nucleus; pm: plasma membrane.

2. Ultrastructural effects of cell incubation with Mel

After the incubation of HaCaT cells with the low dose of Mel (3 µg/mL)—group HM1—the main ultrastructural change noted was the increased number of autophagosomes of 0.2–0.7 µm (Figure S2a-d).

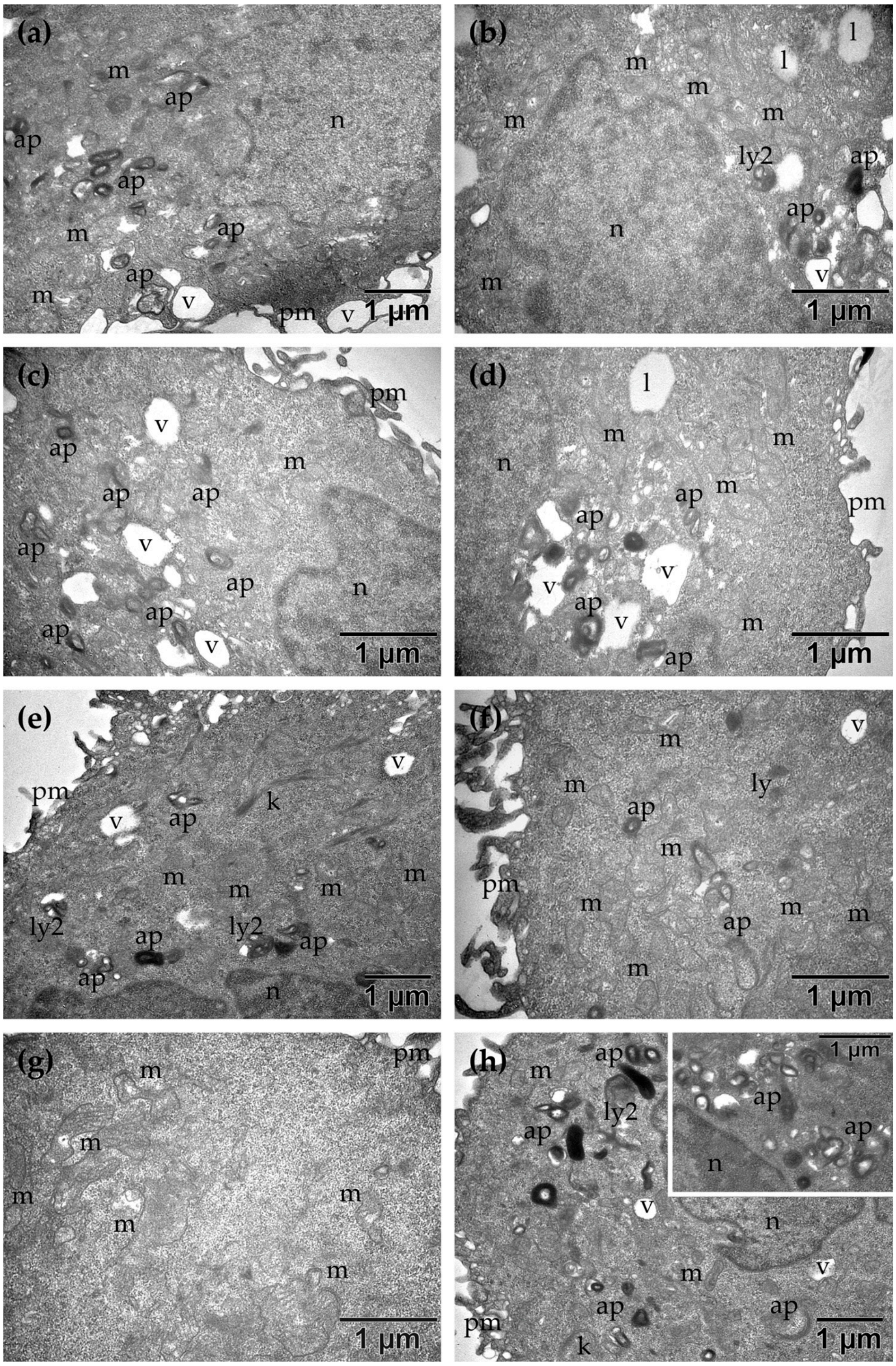

Figure S2: TEM images of cells in the HM1 (a–d) and HM2 (e–h) groups. ap: autophagosome; k: keratin filaments; l: lipid droplets; ly: lysosome; ly2: secondary lysosome; m: mitochondria; n: nucleus; pm: plasma membrane; v: vesicle.

On the other hand, the keratin filaments were rarely present. Most mitochondria preserved a normal structure (Figure S2a-b), but some of them contained electron–lucent areas within the matrix (Figure S2a), while others displayed polymorphous shapes (Figure S2b,d). Lipid droplets had reduced density up to the aspect of empty vacuoles (Figure S2a,c,d). The high dose of Mel (6 μg/mL) triggered the same autophagosomal reaction (increased number and sizes of 0.2–0.6 μm) (Figure S2e,f,h), with many secondary lysosomes also being noted in the group HM2. More keratin bundles were found in the cells of this group (Figure S2e,h). Apart from the many autophagosomes, a second important ultrastructural reaction concerned mitochondria. Almost all were longer (1–1.2 μm) and polymorphic (Figure S2f-h), sometimes with cristae still visible within the granular matrix. Rare lipid droplets were observed, which were most probably transformed into empty, electron–lucent vesicles (Figure S2e,f,h).

The incubation of A375 cells with the low dose of Mel (3 μg/mL)—group AM1—produced important mitochondrial alterations. Firstly, a dramatic and almost generalized reduction in the number of their cristae was observed (Figure S3a-d). Next, many mitochondria presented more or less extensive electron–lucent regions within the rarefied matrices (Figure S3a-d). Finally, a large number of mitochondria were very long (2–2.6 μm) and/or very polymorphic (Figure S3a-d). Autophagosomes were in low numbers, but primary and secondary lysosomes were abundant (Figure S3a,b,d). Another organelle affected by the Mel treatment was the endoplasmic reticulum, presenting enlarged profiles (Figure S3a-c). The rare lipid droplets identified had increased density and extremely diverse shapes (inset of Figure S3d).

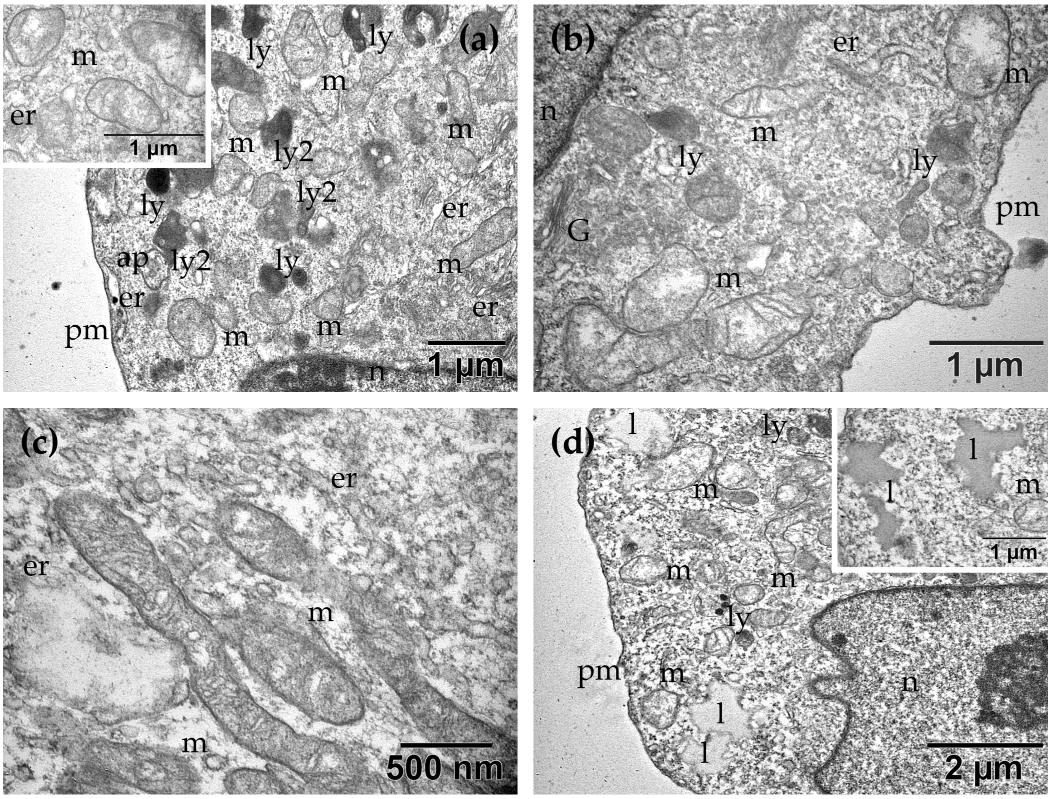

Figure S3: TEM images of cells in the AM1 group. ap: autophagosome; er: endoplasmic reticulum; l: lipid droplets; ly: lysosome; ly2: secondary lysosome; m: mitochondria; n: nucleus; pm: plasma membrane.

3. Ultrastructural Effects of Cell Incubation with MNPs

During the 24 h of incubation with the small dose of MNPs (50 μg/mL), the HaCaT cells in group HMNPs1 internalized large amounts of MNPs. The MNPs were found

inside large or very large intact endosomes (Figure S4a,b,d), proving that the endocytosis process was still ongoing at the moment of fixation. Also, a reduced number of MNPs were visible inside small lysosomes (Figure S4b), while most internalized MNPs were dispersed freely in the cytosol (Figure S4a-d). The general ultrastructure of the cells was unchanged, apart from the presence of many keratin bundles (Figure S4a-d), and the organelles, including mitochondria, displayed a normal aspect. Very rare autophagosomes were found in this group. The incubation with the high dose of MNPs (100 μg/mL)—group HMNPs2—also resulted in the internalization of high amounts of MNPs, visible in endosomes of various sizes (Figure S4e-g), as well as in small lysosomes (Figure S4f), and free in the cytosol (Figure S4e-h). A certain number of secondary lysosomes was noted in this group, and the other organelles showed normal structure. Keratin bundles were also numerous (Figure S4f-h).

The 24 h incubation of the A375 cells with the small dose of MNPs (50 μg/mL)—group AMNPs1—was followed by a moderate internalization of nanoparticles. They were visible inside rare low-to-medium-sized intact endosomes (Figure S5a), inside numerous small lysosomes (Figure S5b), and mostly grouped or dispersed within the cytosol (Figure S5c,d). The general ultrastructure of cells was slightly changed: the endoplasmic reticulum was enlarged (Figure S5a) and some mitochondria had a rarefied matrix (Figure S5d). None or very few endosomes were found, and lipid droplets had reduced density and irregular outlines (Figure S5b,c). Following incubation with the high dose of MNPs (100 μg/mL), the A375 cells apparently internalized higher amounts of MNPs, visible in large (Figure S5e) or small (Figure S5f) endosomes and in small lysosomes (Figure S5f,h), and free in the cytosol (Figure S5f-h). The cells contained a moderate number of small autophagosomes (Figure S5f,g); rare mitochondria were longer (Figure S5h), the lipid droplets had reduced density and irregular outline (Figure S5e,f), and a few electron-lucent vesicles were noted (Figure S5f,g).

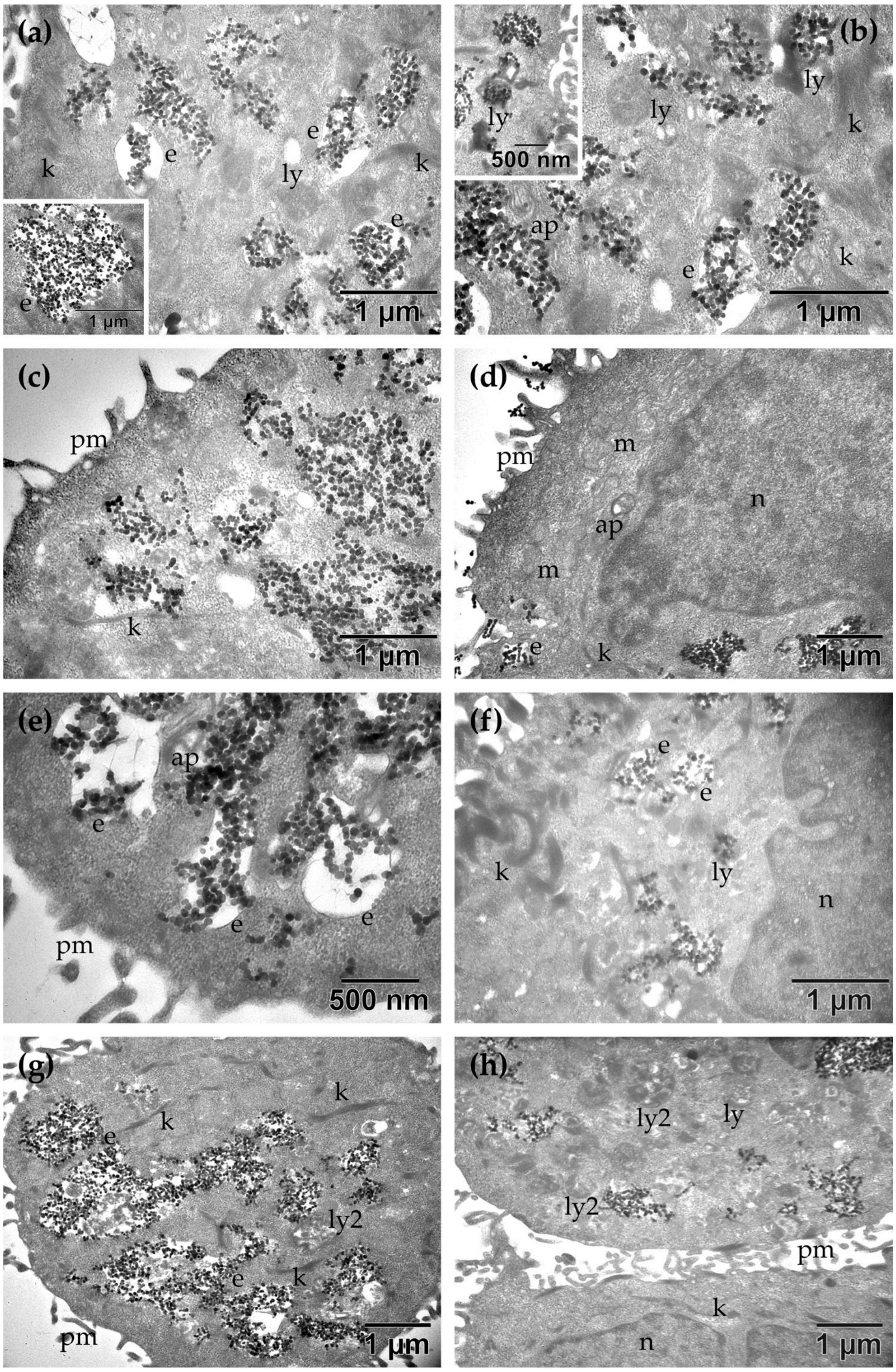

Figure S4. TEM images of the HMNPs1 (a–d) and HMNPs2 (e–h) groups. ap: autophagosome; e: endosome; k: keratin filaments; ly: lysosome; ly2: secondary lysosome; m: mitochondria; n: nucleus; pm: plasma membrane.

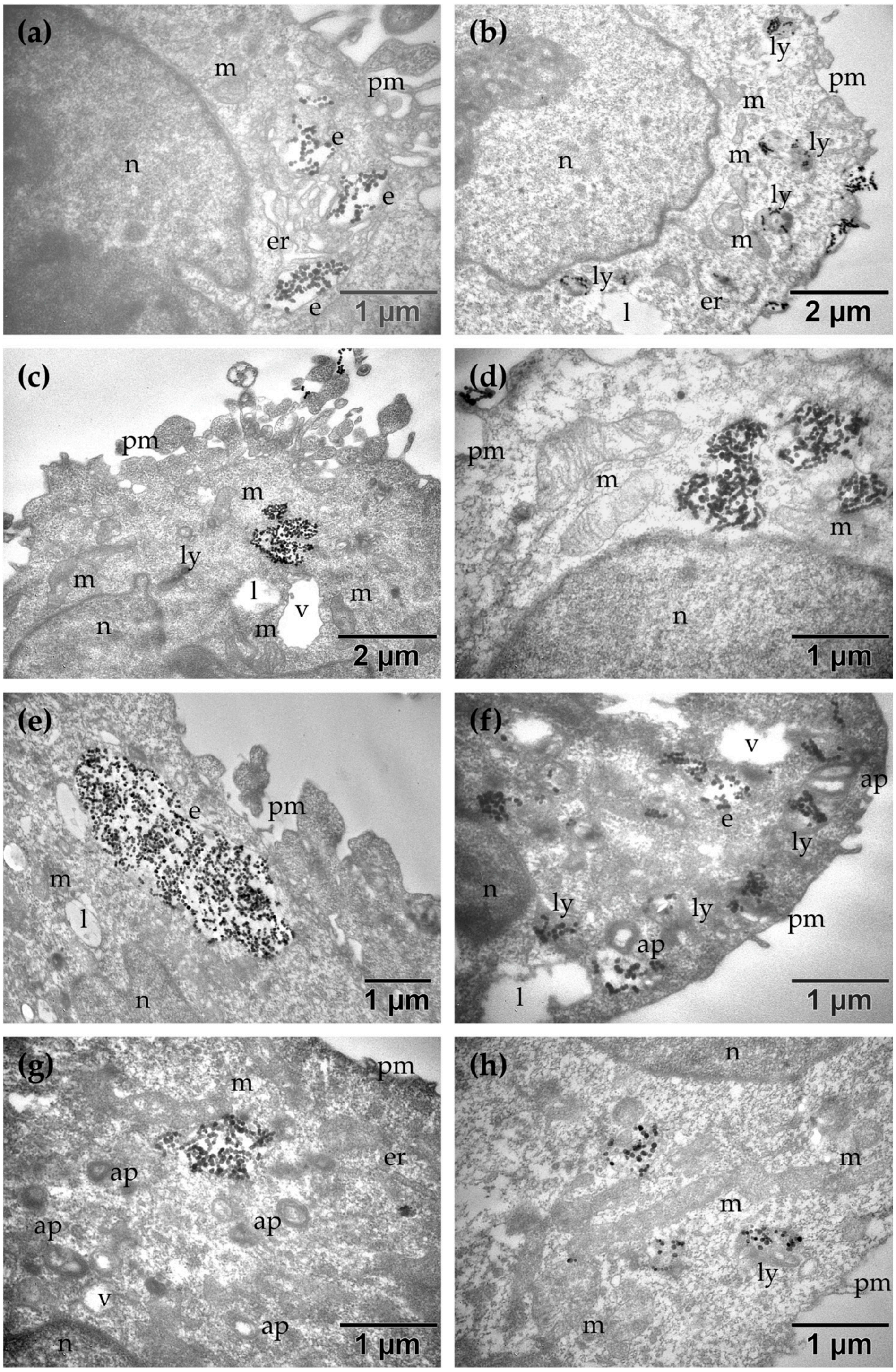

Figure S5. TEM images of the AMNPs1 (a–d) and AMNPs2 (e–h) groups. ap: autophagosome; e: endosome; er: endoplasmic reticulum; l: lipid droplets; ly: lysosome; ly2: secondary lysosome; m: mitochondria; n: nucleus; pm: plasma membrane; v: vesicle.

4. Ultrastructural Effects of Incubation with MNPs and MH Exposure

In the HaCaT cells incubated with the low dose of MNPs (50 µg/mL) and exposed to 30 min MH—group HMNPs1-MH—there were abundant internalized MNPs, mostly inside endosomes (Figure S6a-d), but they were also noted inside lysosomes or free in the cytoplasm (Figure S6a-d). The mitochondria in these cells were long and polymorphic, with a dense, granular matrix and few cristae (Figure S6a,c). Many large lysosomes were observed (Figure S6a-d), as well as occasional thick bundles of keratin fibers (Figure S6d). In the HaCaT cells incubated with a high dose of MNPs (100 µg/mL) and exposed to 30

min MH—group HMNPs2-MH—a high number of internalized MNPs were identified inside endosomes, inside lysosomes, or free in the cytoplasm (Figure S6e-h). The second treatment also generated long and polymorphic mitochondria with rarefied matrix and fewer cristae (Figure S6e-h). Numerous large lysosomes were observed (Figure S6e-h), as well as keratin bundles (Figure S6e,f,h). None or very few autophagosomes were found in these two groups.

In the A375 cells incubated with the low dose of MNPs (50 μg/mL) and exposed to 30 min MH—group AMNPs1-MH—clusters of internalized MNPs were noted inside endosomes of various sizes, including very large ones (Figure S7a,d), in lysosomes (Figure S7b-d) and free in the cytoplasm (Figure S7a,c,d). Most mitochondria were long, polymorphic, and profoundly altered, having a heterogeneous matrix, with electron–lucent regions, and few cristae, some disorganized, with aberrant shapes (Figure S7a,c). Numerous primary and secondary lysosomes were also present (Figure S7a-d), some having irregular, elongated shapes (Figure S7b-d).

In the A375 cells incubated with the high dose of MNPs (100 μg/mL) and exposed to 30 min MH—group AMNPs2-MH—a lower amount of internalized MNPs was found inside endosomes (Figure S7e) or primary and secondary lysosomes (Figure S7e-g). Very few cells with MNPs dispersed in the cytosol were found. However, the most relevant ultrastructural change for this group concerned mitochondria. Most of them were polymorphic, or even with bizarre shapes, very long (2–3.5 μm), of large and uneven diameters (0.5–2 μm), and profoundly altered, having a heterogeneous matrix, with electron–lucent regions and fewer and disorganized cristae (Figure S7e-h). Numerous lysosomes were also present (Figure S7e-g), as well as several autophagosomes of various sizes (Figure S7h). The endoplasmic reticulum was expanded in some cells (Figure S7f), while in others it had a normal aspect (Figure S7h).

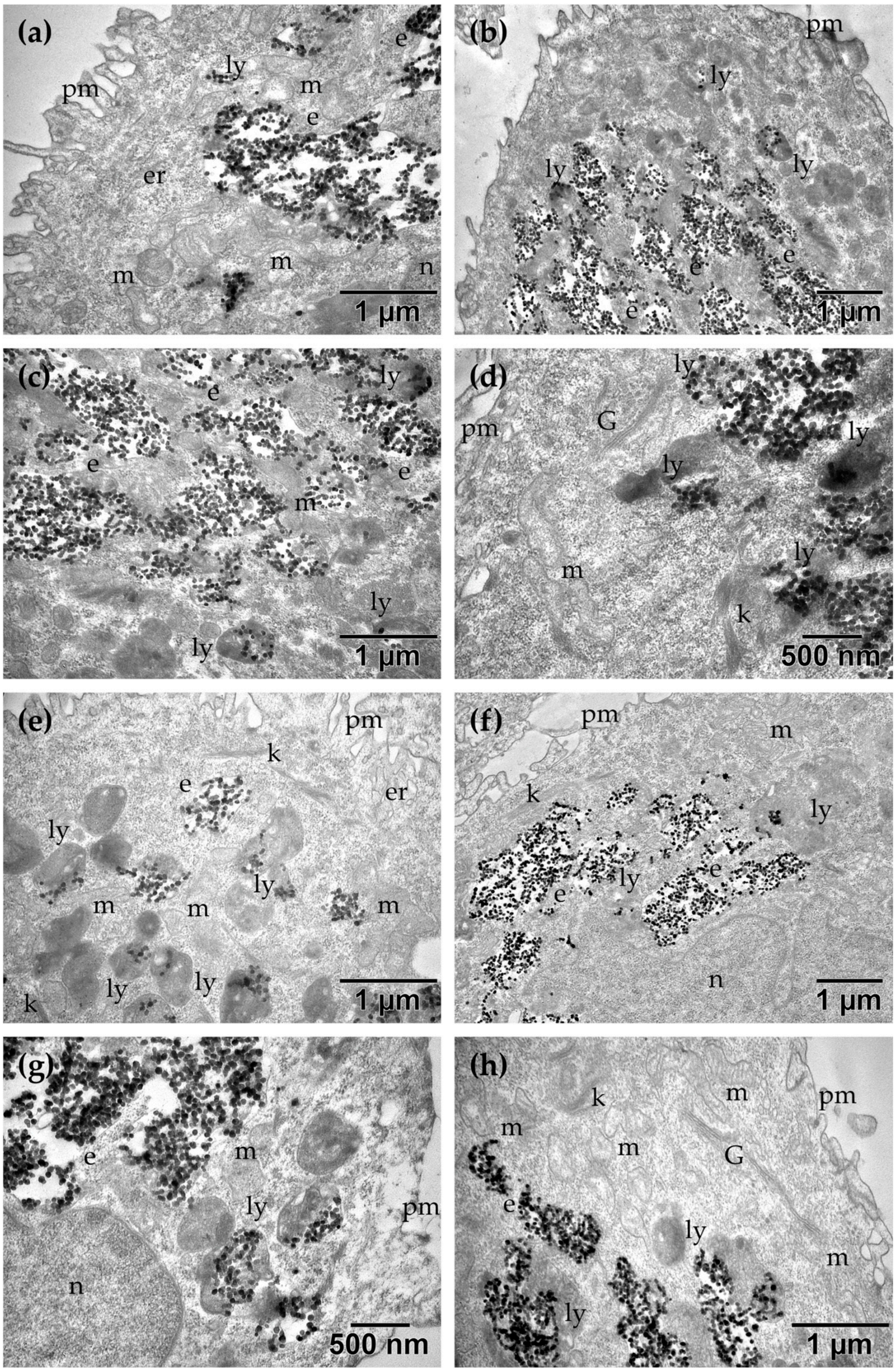

Figure S6. TEM images of the HMNPs1-MH (a-d) and HMNPs2-MH (e–h) groups. e: endosome; er: endoplasmic reticulum; G: Golgi apparatus; k: keratin filaments; ly: lysosome; m: mitochondria; n: nucleus; pm: plasma membrane.

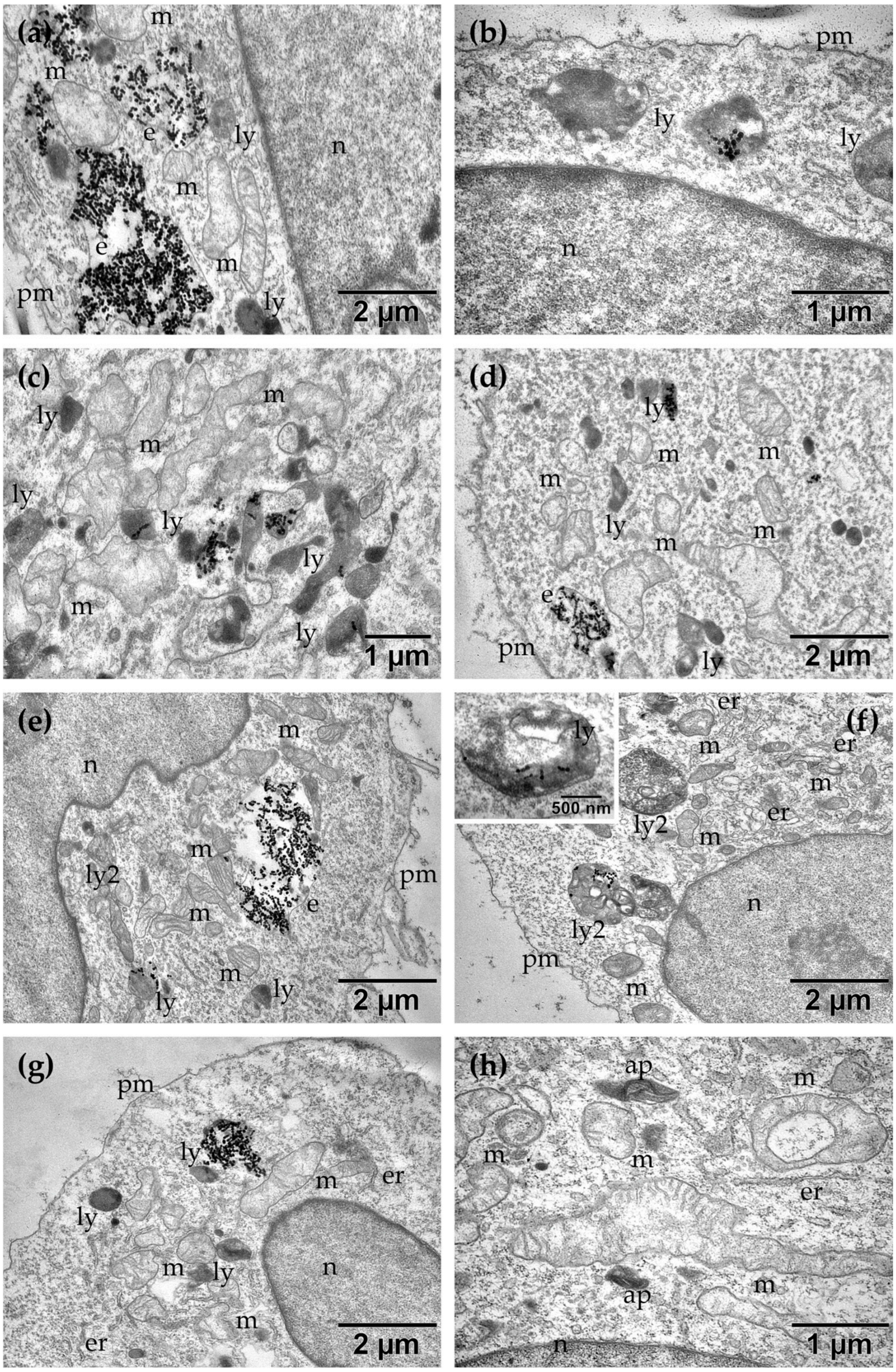

Figure S7. TEM images of the AMNPs1-MH (a–d) and AMNPs2-MH (e–h) groups. ap: autophagosome; e: endosome; er: endoplasmic reticulum; ly: lysosome; ly2: secondary lysosome; m: mitochondria; n: nucleus; pm: plasma membrane.

5. Ultrastructural Effects of Incubation with MNPs-Mel

The HaCaT cells incubated with the low dose of MNPs-Mel (50 μg/mL) internalized them in great numbers inside endosomes (Figure S8a-d), with a low number of MNPs-Mel also being found inside lysosomes (Figure S8a,b). However, the largest amount of MNPs-Mel was observed as dispersed particles within the cytosol (Figure S8a-d). Some mitochondria were polymorphic, with dense matrix (Figure S8b-d), and several lysosomes (Figure S8a,b) and small autophagosomes (Figure S8d) were observed. Additionally, vesicles were present in some cells (Figure S8b,c). In the HaCaT cells incubated with the large

dose of MNPs-Mel (100 μg/mL), the MNPs-Mel were also internalized in great numbers. They were found inside endosomes (Figure S8e-g) or lysosomes (Figure S8e-g), as well as free in the cytoplasm (Fig S8e-g). Mitochondria also reacted by changing shapes and containing a dense matrix (Figure S8f,h), while lysosomes (Figure S8e-h) had a normal aspect. A low number of small autophagosomes or no autophagosomes were observed. The same situation concerned small electron–lucent vesicles (Figure S8h).

In the A375 cells incubated with the low dose of MNPs-Mel (50 μg/mL), only relatively few internalized nanoparticles were found, mainly inside endosomes (Figure S9a), occasionally inside lysosomes (Figure S9b), and rarely dispersed within the cytosol (Figure S8c). In many cells, no MNPs-Mel were noted. Some cells contained ballooned or polymorphic mitochondria, with heterogeneous matrices, containing electron–lucent regions, and few cristae (Figure S9a,c,d), while others had dense matrices and were almost completely devoid of cristae (Figure S9c). Several small, heterogeneous lysosomes were also noted (inset of Figure S9b), as well as rare lipid droplets with irregular contours (Figure S9a,d). Additionally, the autophagosomes were small and in low numbers (Figure S9c). The incubation with the high dose of MNPs-Mel (100 μg/mL) resulted in profound alterations of the A375 cells, with only some of them still having intact plasma membranes (Figure S9e). However, these cells contained fewer organelles, with expansion of the endoplasmic reticulum. The other cells of this group were profoundly altered by the experimental treatment, having rarefied cytoplasm, mitochondria with electron–lucent matrix, lipid droplets and autophagosomes still visible, and areas in which the plasma membrane appeared interrupted (Figure S9f).

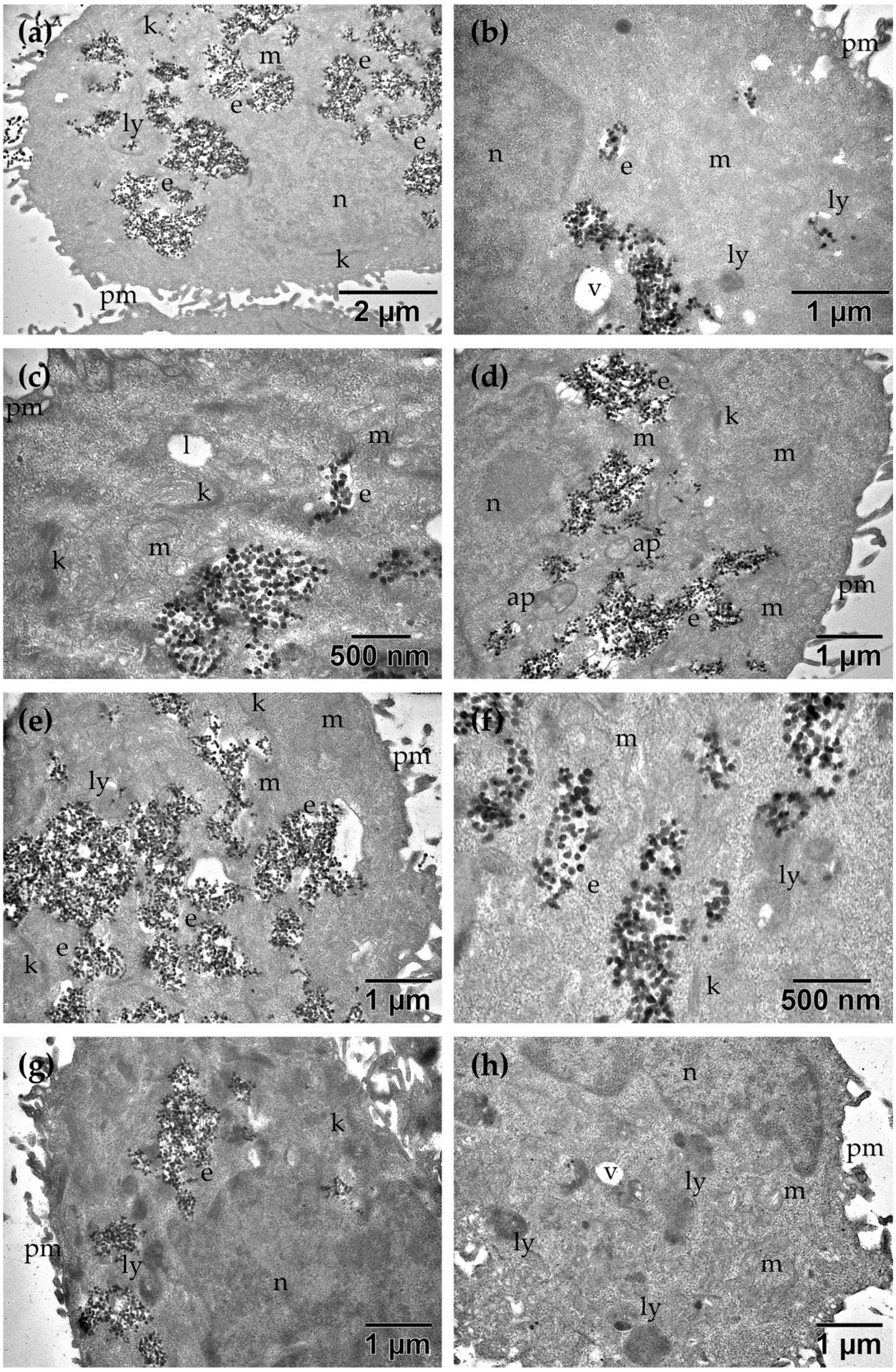

Figure S8. TEM images of the HMNPs-Mel1 (a–d) and HMNPs-Mel2 (e–h) groups. ap: autophagosome; e: endosome; k: keratin filaments; l: lipid droplet; ly: lysosome; m: mitochondria; n: nucleus; pm: plasma membrane; v: vesicle.

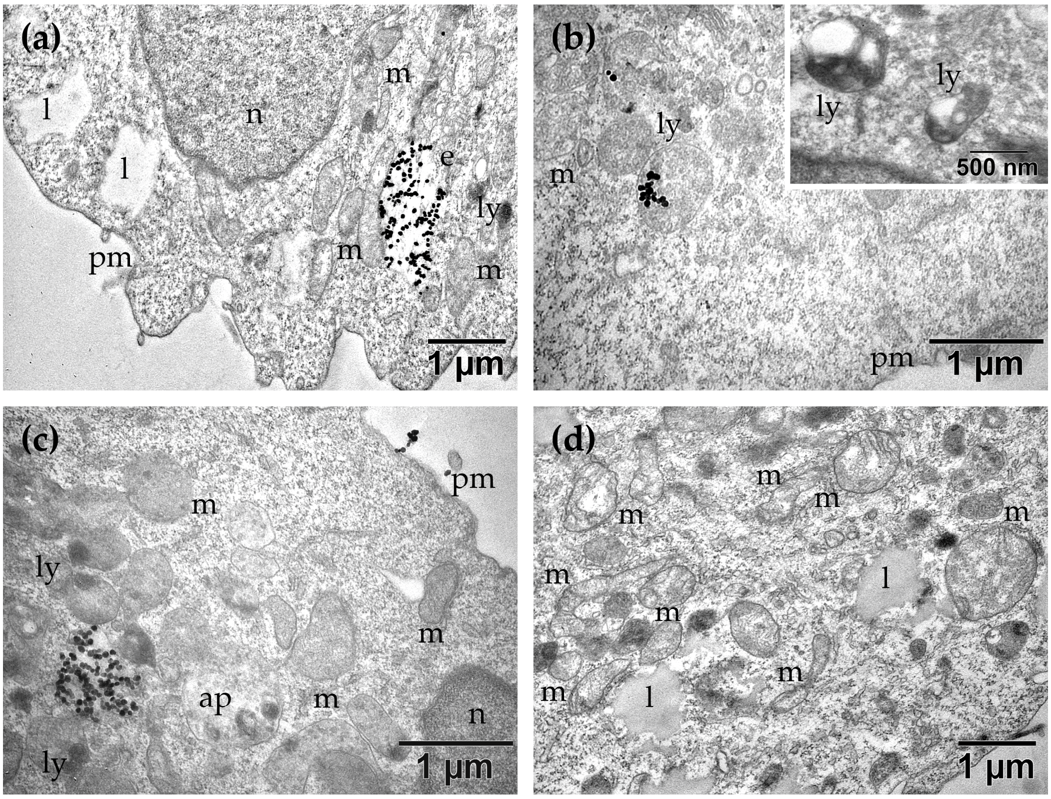

Figure S9. TEM images of cells in the AMNPs-Mel1 group. ap: autophagosome; e: endosome; er: endoplasmic reticulum; l: lipid droplet; ly: lysosome; m: mitochondria; n: nucleus; pm: plasma membrane.

6. Ultrastructural Effects of Incubation with MNPs-Mel and MH Exposure

The HaCaT cells incubated with the low dose of MNPs-Mel (50 µg/mL) and exposed to 30 min of MH—group HMNPs-Mel1-MH—contained abundant internalized MNPs-Mel, found inside large (Figure S10a,b) or small endosomes (Figure S10c,d), inside lysosomes (Figure S10a-d) or free in the cytoplasm (Figure S10a-c). Almost all mitochondria were polymorphic, with either dense and granular or electron–lucent matrix and few cristae (Figure S10a-d). Several relatively large autophagosomes were observed (Figure S10a,c), alongside numerous primary and secondary lysosomes (Figure S10a-d). The HaCaT cells incubated with the high dose of MNPs-Mel (100 µg/mL) and exposed to 30 min MH—group HMNPs-Mel2-MH—contained lower amounts of internalized MNPs-Mel, located in medium-sized or small endosomes (Figure S10e), in numerous lysosomes (Figure S10e-h) or free in the cytoplasm (Figure S10e-h). The cells in this group also had polymorphic mitochondria with either dense, granular matrix or rarefied matrix, in both cases with fewer cristae (Figure S10e,g,h). Large autophagosomes (of 1–1.2 µm) were noted here as well (Figure S10,f), and numerous lysosomes, with different aspects (Figure S10e-h), some containing extensive electron–lucent areas (lower inset in Figure S10g). Additionally, the lipid droplets had reduced density and irregular outline (Figure S10h).

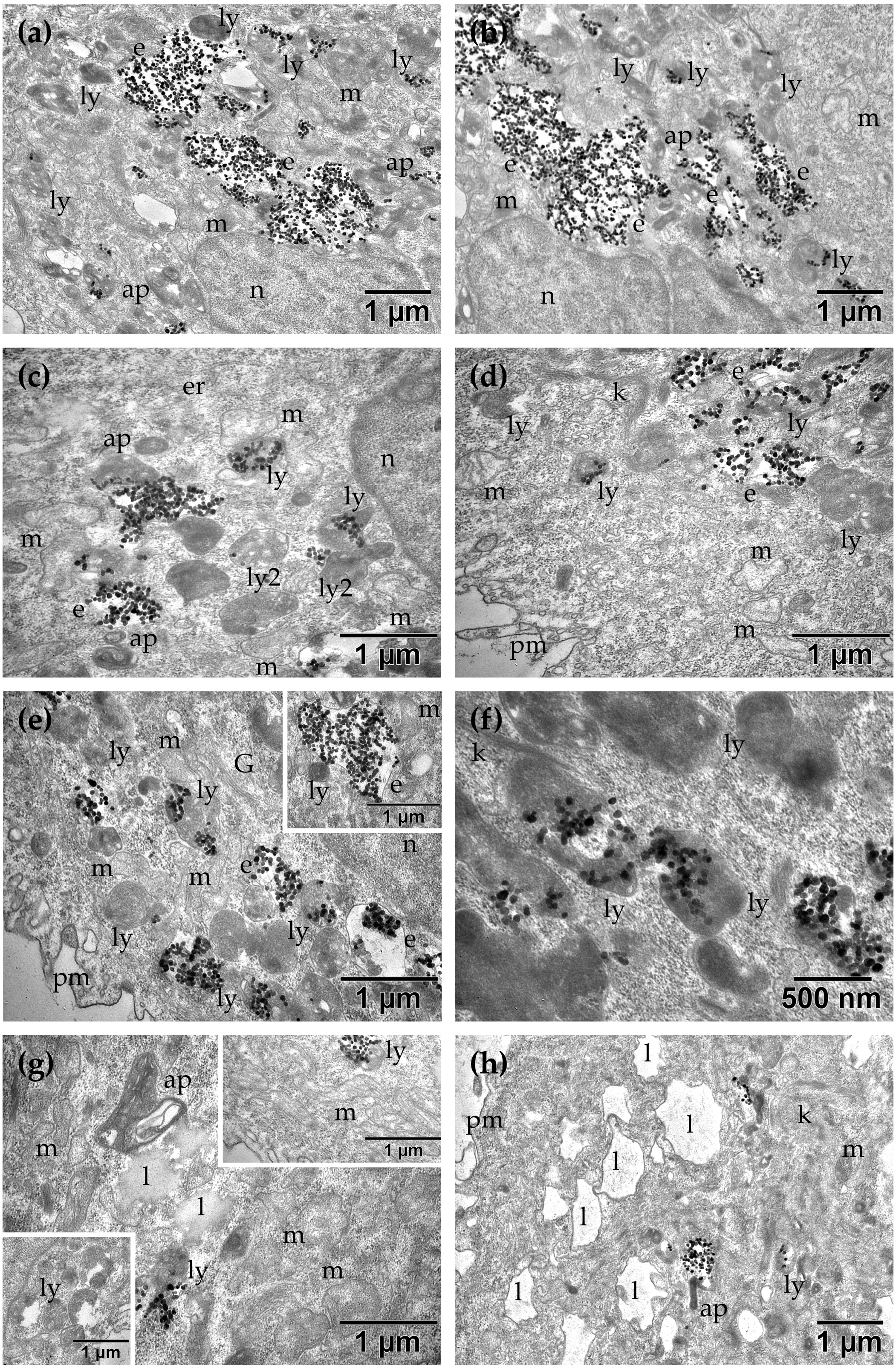

Figure S10. TEM images of cells in the HMNPs-Mel1-MH (a–d) and HMNPs-Mel2-MH (e–h) groups. ap: autophagosome; e: endosome; er: endoplasmic reticulum; G: Golgi apparatus; k: keratin filaments; l: lipid droplet; ly: lysosome; m: mitochondria; n: nucleus; pm: plasma membrane.
